# Risk factors for esophageal squamous cell carcinoma and its histological precursor lesions in China: a multicenter cross-sectional study

**DOI:** 10.1186/s12885-021-08764-x

**Published:** 2021-09-16

**Authors:** Chen Niu, Yong Liu, Jialin Wang, Yuqin Liu, Shaokai Zhang, Yongzhen Zhang, Liwei Zhang, Deli Zhao, Fugang Liu, Lina Chao, Xinzheng Wang, Chunli Zhang, Guohui Song, Zhiyi Zhang, Youpeng Li, Zheng Yan, Yongxiu Wen, Yinyin Ge, Zhaoping Zang, Wei Feng, Haiping Zhang, Lixin Tao, Rena Nakyeyune, Yi Shen, Yi Shao, Xiuhua Guo, Toni Miles, Aiming Yang, Fen Liu, Guiqi Wang

**Affiliations:** 1 Department of Epidemiology and Health Statistics, School of Public Health, Beijing Municipal Key Laboratory of Clinical Epidemiology, Capital Medical University, Beijing, 100069 China; 2 Department of Endoscopy, National Cancer Center/National Clinical Research Center for Cancer/Cancer Hospital, Chinese Academy of Medical Sciences and Peking Union Medical College, Beijing, 100021 China; 3 Shandong Cancer Hospital and Institute, Shandong First Medical University and Shandong Academy of Medical Sciences, Shandong, 250000 China; 4 Gansu Provincial Cancer Hospital, Gansu, 730000 China; 5 Department of Cancer Epidemiology and Prevention, The Affiliated Cancer Hospital of Zhengzhou University, Henan Cancer Hospital, Henan, 450008 China; 6 Department of Epidemiology, Shanxi Cancer Hospital, Shanxi, 030013 China; 7 The Fourth Hospital of Hebei Medical University, Hebei, 050000 China; 8 Feicheng People’s Hospital, Shandong, 271600 China; 9 Dongping People’s Hospital, Shandong, 271500 China; 10 Department of Epidemiology, Hebi People’s Hospital, Henan, 458030 China; 11 Yangcheng Cancer Hospital, Shanxi, 048100 China; 12 The First People’s Hospital of Ningyang County, Shandong, 271400 China; 13 Cixian Institute for Cancer Prevention and Control, Hebei, 056500 China; 14 Gansu Wuwei Cancer Hospital, Gansu, 733000 China; 15 Minqin County People’s Hospital, Gansu, 733000 China; 16 Linze County People’s Hospital, Gansu, 734200 China; 17 Shandan County People’s Hospital, Gansu, 734000 China; 18 Gaotai County People’s Hospital, Gansu, 734300 China; 19 Department of Epidemiology and Biostatistics, College of Public Health, University of Georgia, Athens, GA USA; 20 Department of Gastroenterology, Peking Union Medical College Hospital, Chinese Academy of Medical Sciences, Beijing, 100730 China

**Keywords:** Esophageal squamous cell carcinoma, Precancerous lesions, Cross-sectional study, Risk factor, Prevention

## Abstract

**Background:**

Despite research efforts, the causative factors that contribute to esophageal squamous cell carcinoma (ESCC) in high-risk areas have not yet been understood. In this study, we, therefore, aimed to describe the risk factors associated with ESCC and its precursor lesions.

**Methods:**

We performed an endoscopic examination of 44,857 individuals aged 40–69 years from five high incidence regions of China in 2017–2018. Participants were classified as 4 groups of normal control, esophagitis, low-grade intraepithelial neoplasia (LGIN) and high-grade intraepithelial neoplasia/esophageal squamous cell carcinoma (HGIN/ESCC) using an unconditional logistic regression determine risk factors.

**Results:**

We identified 4890 esophagitis, 1874 LGIN and 437 HGIN/ESCC cases. Crude odds ratios (ORs) and adjusted odds ratios were calculated using unconditional logistic regression. Drinking well and surface water, salty diet, and positive family history of cancer were the common risk factors for esophagitis, LGIN and HGIN/ESCC. History of chronic hepatitis/cirrhosis was the greatest risk factor of esophagitis (adjusted OR 2.96, 95%CI 2.52–3.47) and HGIN/ESCC (adjusted OR 1.91, 95%CI 1.03–3.22). Pesticide exposure (adjusted OR 1.20, 95%CI 1.05–1.37) was essential risk factor of LGIN.

**Conclusions:**

Among individuals aged 40–69 years in high incidence regions of upper gastrointestinal cancer, the results provided important epidemiological evidence for the prevention of different precancerous lesions of ESCC.

**Supplementary Information:**

The online version contains supplementary material available at 10.1186/s12885-021-08764-x.

## Background

Esophageal cancer ranks seventh in terms of incidence and sixth in mortality overall according to the report of the 2018 Global Cancer Statistics [1]. In China, esophageal cancer is the sixth most frequent malignant tumor and the fourth leading cause of cancer-related death [2]. Esophageal adenocarcinoma (EADC) and esophageal squamous cell carcinoma (ESCC) are the two most common histologic subtypes of esophageal cancer [3]. In high-income countries, majority of the esophageal cancer patients have EADCs. In China, more than 90% of the esophageal cancer cases are ESCCs [4]. The most studied region of China is located in the North Central Taihang Mountain. In this area, ESCC is the leading cause of death [5]. Based on a recent report from this region, esophageal cancer prognosis is poor and the 5-year survival rates are about 30% [6].

Esophagitis is usually a benign disease. However, it is associated with an elevated risk for subsequent ESCC. Based on the WHO tumor classification, endoscopic biopsy of precancerous lesions yield low-grade intraepithelial neoplasia (LGIN) or high-grade intraepithelial neoplasia (HGIN) [7]. A report using a sample of containing both lesion types reported a 74% risk for esophageal cancer [8]. Identification of precursor lesions is critical for clinical care because these precursor lesions are curable. Early identification can reduce or delay ESCC mortality [9]. Overall, epidemiologic studies of ESCC show that it is population-dependent. The multifactorial basis for ESCC includes environmental exposures, lifestyle factors, and genetic traits [10]. In Western countries, alcohol consumption and smoking are the major risk factors for ESCC leading to a sevenfold increased risk of disease for smokers [11]. In high-risk areas of China, tobacco or alcohol does not appear to play the same role in population level rates of ESCC [12, 13]. Epidemiologic studies in these areas have not identified alternatives to smoking and alcohol consumption to explain these high rates of ESCC [14]. By increasing the number of cases of different esophageal lesions, this report presents evidence to improve our understanding of population-level factors in high rate areas of China.

To fill the gap in our understanding of risk factors at different stages of esophageal lesions - esophagitis, LGIN and HGIN - this report presents cross-sectional data from five high-incidence regions of ESCC in China.

## Methods

### Study population

This was a multicenter cross-sectional study, which relies on the high incidence regions of esophageal, gastric and colorectal cancer established by the cancer early diagnosis and early treatment project in China [15]. In 2017, we launched a new screening study of upper gastrointestinal cancer in five high-risk regions of upper gastrointestinal cancer in China, including Hebei, Henan, Shandong, Shanxi, and Gansu Provinces. The main purpose of this project is to identify the high-risk population of malignant upper gastrointestinal cancer and to establish a cancer risk prediction model to provide support for the prevention of upper gastrointestinal cancer.

**Fig. 1:**
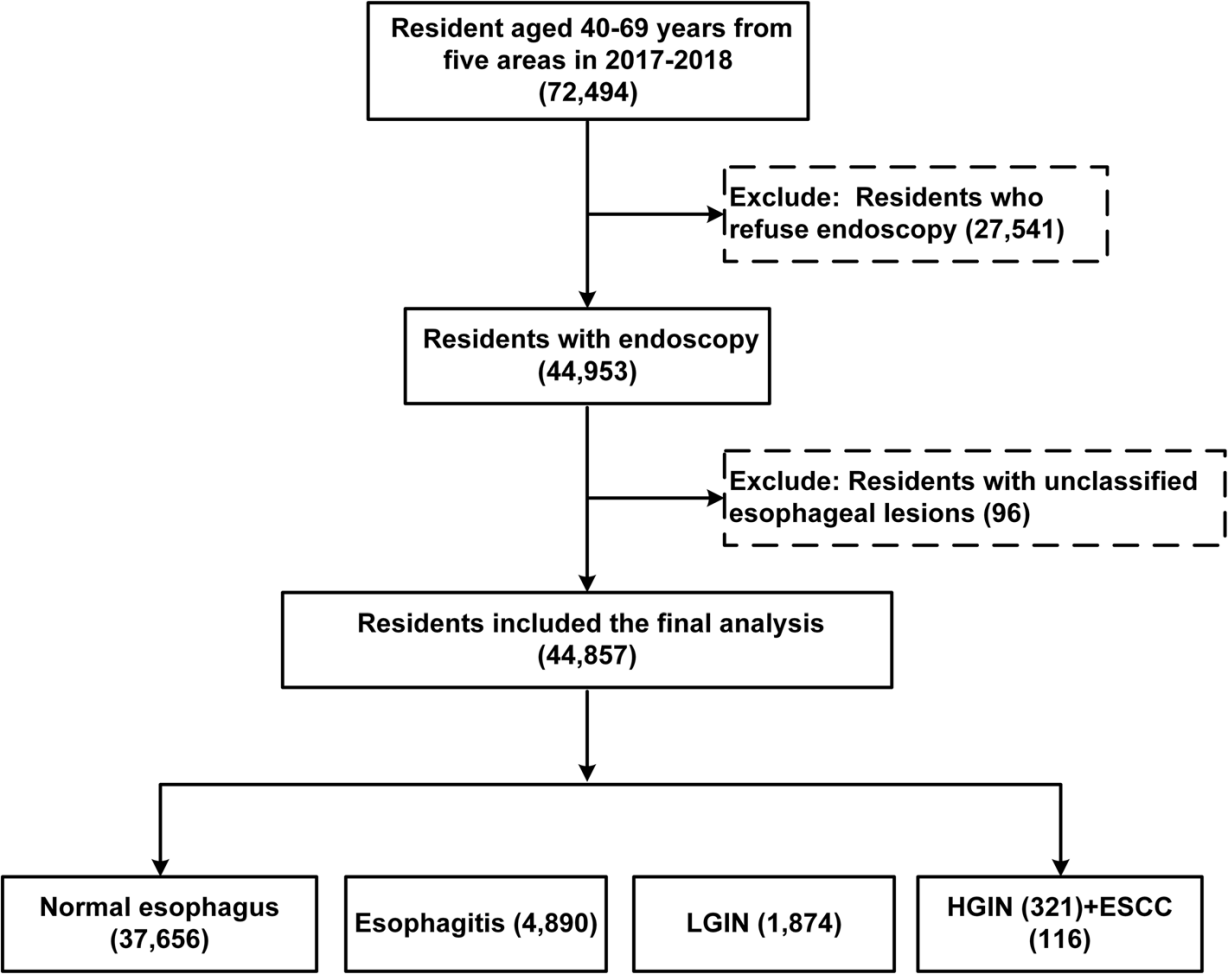
Flow chart of participant selection. LGIN = low-grade intraepithelial neoplasia; HGIN = high-grade intraepithelial neoplasia; ESCC = esophageal squamous cell carcinoma

The inclusion criteria were as follows: (1) local permanent residents in selected regions, (2) no history of endoscopic examination during the last 3 years, (3) no history of cancer, mental disorder, or any contraindication for endoscopy, (4) signed informed consent, and (5) agreement to complete all survey and examination, including endoscopy. The participant selection process is shown in Fig. 1. We recruited participants from April 2017 to December 2018. The final analysis included 44,857 residents aged 40–69 years. Among these participants, there were 116 invasive ESCC cases, 321 HGIN cases, 1874 LGIN cases, 4890 esophagitis cases, and 37,656 normal esophagus controls.

The study was approved by the Capital Medical University, Chinese Academy of Medical Sciences and Peking Union Medical College. All the experiment protocol for involving humans was in accordance to guidelines of Declaration of Helsinki. The STROBE statement can be found in Additional File 1.

### Survey and measurement

Comprehensive questionnaire information was collected by face-to-face interviews and entered directly into a laptop based data entry system by trained investigators. The data entry process was facilitated with software designed to decrease missing items and reduce logic inaccuracy. A full questionnaire commonly took 35 ~ 45 min to accomplish. Items in the survey included demographic characteristics (e.g. gender, age), socio-economic status (e.g. occupation, education and income), lifestyle habits (e.g. smoking, alcohol and tea drinking), eating habits (e.g. fresh fruit, meat and milk), history of diseases (e.g. history of chronic hepatitis/cirrhosis), as well as medical history (e.g. acid suppressant medications and antibiotics). In this study, occupation and education level were also classified two categories: farmer/non-farmer and formal/no formal. In addition, frequency of consumption of specific dietary components was collected using an item with a five-level response category. The responses included: eating every day, 4–6 days per week, 1–3 days per week, 1–3 days per month and never eating. Body mass index (BMI) was calculated by the formula: weight (kg)/height (m^2^) and then classified as underweight if BMI < 18.5 kg/m^2^, normal if between 18.5 kg/m^2^ and 23.9 kg/m^2^, overweight if between 24.0 kg/m^2^ and 27.9 kg/m^2^, and obese if BMI ≥ 28.0 kg/m^2^ according to the Chinese population recommendation [16]. The detailed definitions of the variables were shown in Additional File 2.

The endoscopic examinations were conducted by physicians at local hospitals. Procedures were based on clinical guidelines for cancer screening and early diagnosis and treatment in China [17]. The whole esophagus was visually examined. Lugol’ iodine staining in esophagus was applied to the diagnosis of suspicious lesions. The subjects with suspicious lesions (unstained and inflammatory lesions) were needed biopsy. The number of biopsies depended on the size of the lesion (> 1 cm, ≥ 2 biopsy; > 2 cm, ≥ 3 biopsy; > 3 cm, ≥ 4 biopsy). In addition, if multiple scattered lesions were found, biopsy of all suspicious lesions should be taken as much as possible. Subjects without suspicious lesions did not receive a biopsy. The biopsy slides were read by two pathologists independently. If there were any inconsistencies, a third pathologist would give advice through discussion. Doctors reported the worst biopsy diagnosis to participants with multiple lesions. The histological criteria were as previously described [18–20].

To ascertain severity, esophageal mucosa was ranked using 5 categories: normal esophageal mucosa, minor mucosa changes, esophagitis, esophageal squamous simple hyperplasia (ESSH) or esophageal squamous dysplasia (ESD) [21]. ESD was further categorized into 3 levels including mild, moderate, and severe. According to WHO tumor histological classification [7], mild and moderate ESD combined fall under LGIN. Severe ESD and squamous cell carcinoma in situ (CIS) are considered as the HGIN. In this study, we grouped the participants into 4 groups: normal control, esophagitis, LGIN and HGIN/ESCC (lesions including severe ESD, CIS, and higher-grade lesions).

### Statistical analyses

Basic, descriptive statistics show categorical variables as frequencies and percentages, while continuous variables are shown as mean and standard deviations. The appropriate tests for significance were applied, *χ*^*2*^ or *ANOVA* test, respectively. In this report, we use the unconditional univariable logistic model to calculate crude odds ratios (ORs) and 95% confidence intervals (CIs) [22]. The variables with a *P* value ≤0.2 in unconditional univariable logistic analysis were selected for unconditional multivariable logistic analysis. We considered three models to calculate the adjusted OR, including age-adjusted only, gender-adjusted only and adjusted age, gender, education, BMI and income at the same time. These adjusted confounders were selected based on the previous studies of esophageal cancer. Besides, models of adjustment for age or gender only were conducted as they may influence the socioeconomic status. In this study, dependent variable was the diagnosis outcome of disease. The independent variables included environment, lifestyle, diet habits, and medical history. We used SPSS software (version 24.0) to perform *χ*^*2*^ or *ANOVA* test, and glm function of R software (version 3.6.1) to perform logistic regression analysis. All tests of significance were two-sided with a *P* value of 0.05 was considered as statistically significant, expect univariable logistic regression analysis.

## Results

### Participant characteristics

A total of 44,857 subjects aged 40 to 69 were incorporated in the analysis, including 37,656 (83.95%) subjects with a normal esophagus, 4890 (10.90%) people with esophagitis, 1874 (4.18%) cases of LGIN and 437 (0.97%) cases of HGIN/ESCC. Because the complete statistical description table was too long (Additional File 3), we only selected a few main variables to show in Table 1. The proportion of male in normal group, esophagitis, LGIN and HGIN/ESCC was 41.4, 46.8, 48.7 and 56.5%, respectively. However, the normal group (54.41 ± 7.26) was composed of a slightly younger population than the esophagitis (57.29 ± 6.85), LGIN (59.22 ± 6.25) and HGIN/ESCC (60.85 ± 5.80) group.

**Table 1:** Characteristics of participants and endoscopy results of different esophageal lesions in Chinese high-risk population in 2017–2018

Factors	HGIN/ESCC *n* = 437	LGIN *n* = 1874	Esophagitis *n* = 4890	Norma esophagus *n* = 37,656	Total cohort *n* = 44,857	*P* value
**Gender, n(%)**						< 0.001^a^
Male	247 (56.5)	913 (48.7)	2290 (46.8)	15,590 (41.4)	19,040 (42.4)	
Female	190 (43.5)	961 (51.3)	2600 (53.2)	22,066 (58.6)	25,817 (57.6)	
**Age, mean (SD)**	60.85 (5.80)	59.22 (6.25)	57.29 (6.85)	54.41 (7.26)	54.99 (7.30)	< 0.001^b^
**Education, n(%)**						< 0.001^a^
No formal	81 (18.5)	368 (19.6)	744 (15.2)	5395 (14.3)	6588 (14.7)	
Formal	356 (81.5)	1506 (80.4)	4146 (84.8)	32,261 (85.7)	38,269 (85.3)	
**Occupation, n(%)**						< 0.001^a^
Non-farmer	86 (19.7)	382 (20.4)	1028 (21.0)	9347 (24.8)	10,843 (24.2)	
Farmer	351 (80.3)	1492 (79.6)	3862 (79.0)	28,309 (75.2)	34,014 (75.8)	
**BMI (kg/m**^**2**^**), n(%)**						< 0.001^a^
< 18.5	16 (3.7)	36 (1.9)	98 (2.0)	714 (1.9)	864 (1.9)	
18.5 to 23.9	229 (52.3)	914 (48.8)	2336 (47.8)	17,309 (46.0)	20,788 (46.3)	
24.0 to 27.9	159 (36.4)	742 (39.6)	1977 (40.4)	15,074 (40.0)	17,952 (40.1)	
≥ 28.0	33 (7.6)	182 (9.7)	479 (9.8)	4559 (12.1)	5253 (11.7)	

Source: WHO tumor histological classification 2000. ^a^ = *χ*^*2*^ test; ^b^ = *ANOVA* test. Population recruited from five high-rate regions for upper gastrointestinal cancer in China, including Hebei, Henan, Shandong, Shanxi, and Gansu Provinces

*BMI* body mass index (kg/m^2^), *LGIN* low-grade intraepithelial neoplasia, *HGIN* high-grade intraepithelial neoplasia, *ESCC* esophageal squamous cell carcinoma

### Risk factors for different esophageal lesions

We applied two steps to identify risk factors. Firstly, the unconditional univariable logistic regression analysis was used to test each variable independently and the estimated ORs with 95%CI are listed in Additional File 4. Secondly, the statistically significant variables in unconditional univariable logistic analysis were selected for unconditional multivariable analysis and the results were shown in Additional File 5. Tables 2, 3, 4 displayed the significant factors of 3 lesion groups from unconditional univariable logistic analysis and multivariable logistic analysis adjusted for age, gender, education, BMI and income.

**Table 2:** Demographic characteristics associated with different esophageal lesions

Demographic characteristics	HGIN/ESCC				LGIN				Esophagitis			
	OR (95% CI)	*P* value	Adjusted OR ^a^ (95% CI)	*P* value	OR (95% CI)	*P* value	Adjusted OR ^a^ (95% CI)	*P* value	OR (95% CI)	*P* value	Adjusted OR ^a^ (95% CI)	*P* value
**Gender (Female)**	0.54 (0.45–0.66)	< 0.001	–	–	0.74 (0.68–0.82)	< 0.001	–	–	0.80 (0.76–0.85)	< 0.001	–	–
**Age, years**												
40–49	1.00 (reference)	–	–	–	1.00 (reference)	–	–	–	1.00 (reference)	–	–	–
50–59	4.96 (3.12–8.40)	< 0.001	–	–	3.17 (2.65–3.81)	< 0.001	–	–	1.80 (1.65–1.96)	< 0.001	–	–
60–69	15.73 (10.06–26.29)	< 0.001	–	–	7.18 (6.04–8.60)	< 0.001	–	–	2.84 (2.60–3.10)	< 0.001	–	–
**Education (Formal)**	0.73 (0.58–0.94)	0.013	–	–	0.68 (0.61–0.77)	< 0.001	–	–	0.93 (0.86–1.01)	0.097	–	–
**Annual income per family (RMB)**												
< 10,000	1.00 (reference)	–	–	–	1.00 (reference)	–	–	–	1.00 (reference)	–	–	–
10,000-	0.65 (0.49–0.89)	0.005	–	–	1.00 (0.86–1.18)	0.973	–	–	0.86 (0.78–0.95)	0.005	–	–
30,000-	0.80 (0.61–1.07)	0.124	–	–	1.03 (0.88–1.20)	0.725	–	–	1.02 (0.93–1.13)	0.564	–	–
50,000-	0.60 (0.43–0.85)	0.004	–	–	0.82 (0.69–0.99)	0.033	–	–	0.87 (0.78–0.98)	0.018	–	–
≥70,000	0.42 (0.26–0.65)	< 0.001	–	–	0.58 (0.46–0.73)	< 0.001	–	–	0.90 (0.79–1.02)	0.106	–	–
**BMI**												
18.5 to 23.9	1.00 (reference)	–	–	–	1.00 (reference)	–	–	–	1.00 (reference)	–	–	–
< 18.5	1.69 (0.97–2.73)	0.044	–	–	0.95 (0.67–1.32)	0.791	–	–	1.02 (0.82–1.26)	0.878	–	–
24.0 to 27.9	0.80 (0.65–0.98)	0.029	–	–	0.93 (0.84–1.03)	0.166	–	–	0.97 (0.91–1.04)	0.379	–	–
≥ 28.0	0.55 (0.37–0.78)	0.001	–	–	0.76 (0.64–0.89)	0.001	–	–	0.78 (0.70–0.86)	< 0.001	–	–
**Family history of cancer**	1.50 (1.23–1.82)	< 0.001	1.64 (1.34–2.00)	< 0.001	1.24 (1.12–1.37)	< 0.001	1.33 (1.20–1.47)	< 0.001	1.19 (1.11–1.27)	< 0.001	1.22 (1.14–1.30)	< 0.001
**History of chronic hepatitis and cirrhosis**	1.96 (1.07–3.28)	0.018	1.91 (1.03–3.22)	0.025	1.36 (0.96–1.86)	0.067	1.36 (0.96–1.87)	0.070	3.05 (2.60–3.57)	< 0.001	2.96 (2.52–3.47)	< 0.001

Source: WHO tumor histological classification 2000

*BMI* body mass index (kg/m^2^), *LGIN* low-grade intraepithelial neoplasia, *HGIN* high-grade intraepithelial neoplasia, *ESCC* esophageal squamous cell carcinoma

^a^adjusted for age, gender, education, BMI and income

**Table 3:** Environment, lifestyle and habits associated with different esophageal lesions

Environment, lifestyle and habits	HGIN/ESCC				LGIN				Esophagitis			
	OR (95% CI)	*P* value	Adjusted OR ^a^ (95% CI)	*P* value	OR (95% CI)	*P* value	Adjusted OR ^a^ (95% CI)	*P* value	OR (95% CI)	*P* value	Adjusted OR ^a^ (95% CI)	*P* value
**Smoking**												
Not smoke	1.00 (reference)	–	1.00 (reference)	–	1.00 (reference)	–	1.00 (reference)	–	1.00 (reference)	–	1.00 (reference)	–
Former/current smoke	1.97 (1.62–2.41)	< 0.001	1.46 (1.14–1.89)	0.003	1.28 (1.15–1.43)	< 0.001	1.04 (0.91–1.19)	0.574	1.25 (1.16–1.33)	< 0.001	1.09 (1.00–1.19)	0.048
**Occupation (Farmer versus Non-farmer)**	1.35 (1.07–1.72)	0.014	1.22 (0.97–1.57)	0.098	1.29 (1.15–1.45)	< 0.001	1.20 (1.07–1.35)	0.002	1.24 (1.15–1.33)	< 0.001	1.21 (1.12–1.30)	< 0.001
**Pesticide exposure**	1.46 (1.13–1.87)	0.003	1.44 (1.11–1.84)	0.005	1.23 (1.08–1.40)	< 0.001	1.20 (1.05–1.37)	0.007	1.04 (0.95–1.13)	0.435	–	–
**Refrigerator use, length of time**												
No refrigerator	1.00 (reference)	–	1.00 (reference)	–	1.00 (reference)	–	1.00 (reference)	–	1.00 (reference)	–	1.00 (reference)	–
1-10 years	0.57 (0.43–0.78)	< 0.001	0.76 (0.57–1.04)	0.074	0.74 (0.63–0.87)	< 0.001	0.88 (0.75–1.04)	0.135	0.66 (0.60–0.74)	< 0.001	0.73 (0.66–0.81)	< 0.001
11-20 years	0.52 (0.36–0.76)	0.001	0.71 (0.49–1.05)	0.087	0.64 (0.52–0.78)	< 0.001	0.78 (0.64–0.95)	0.015	0.61 (0.54–0.69)	< 0.001	0.67 (0.59–0.77)	< 0.001
> 20 years	0.44 (0.11–1.21)	0.170	0.55 (0.13–1.52)	0.319	0.60 (0.33–1.01)	0.070	0.69 (0.37–1.16)	0.189	0.48 (0.32–0.69)	< 0.001	0.50 (0.33–0.71)	< 0.001
**Alcohol**												
Not drink	1.00 (reference)	–	1.00 (reference)	–	1.00 (reference)	–	1.00 (reference)	–	1.00 (reference)	–	1.00 (reference)	–
Former/current drink	2.15 (1.68–2.75)	< 0.001	1.60 (1.23–2.09)	0.001	1.54 (1.34–1.76)	< 0.001	1.30 (1.12–1.51)	< 0.001	1.22 (1.10–1.34)	< 0.001	1.05 (0.95–1.17)	0.331
**Tea drinking frequency**												
Not drink	1.00 (reference)	–	1.00 (reference)	–	1.00 (reference)	–	1.00 (reference)	–	1.00 (reference)	–	1.00 (reference)	–
Former/current drink	1.35 (1.11–1.65)	0.003	1.32 (1.08–1.61)	0.007	1.43 (1.30–1.57)	< 0.001	1.34 (1.26–1.54)	< 0.001	0.78 (0.73–0.83)	< 0.001	0.76 (0.70–0.81)	< 0.001
**Water source (**Well water and surface water**)**	1.64 (1.35–1.98)	< 0.001	1.55 (1.28–1.89)	< 0.001	1.25 (1.13–1.37)	< 0.001	1.19 (1.08–1.32)	< 0.001	1.59 (1.50–1.69)	< 0.001	1.61 (1.51–1.71)	< 0.001
**Tea temperature**^**b**^												
Warm tea	1.00 (reference)	–	1.00 (reference)	–	1.00 (reference)	–	1.00 (reference)	–	1.00 (reference)	–	1.00 (reference)	–
Hot/burning hot tea	1.34 (0.91–1.99)	0.144	1.34 (0.90–1.99)	0.151	1.11 (0.92–1.33)	0.277	1.10 (0.92–1.33)	0.300	1.10 (0.95–1.27)	0.202	1.12 (0.97–1.29)	0.137
**Drink improved water**	0.74 (0.49–1.08)	0.137	0.79 (0.52–1.15)	0.251	0.56 (0.44–0.69)	< 0.001	0.58 (0.46–0.72)	< 0.001	0.72 (0.64–0.82)	< 0.001	0.73 (0.64–0.83)	< 0.001
**Diet taste**												
Light diet	1.00 (reference)	–	1.00 (reference)	–	1.00 (reference)	–	1.00 (reference)	–	1.00 (reference)	–	1.00 (reference)	–
Salty diet	1.40 (1.09–1.81)	0.009	1.36 (1.07–1.77)	0.016	1.60 (1.41–1.83)	< 0.001	1.57 (1.38–1.80)	< 0.001	1.59 (1.47–1.74)	< 0.001	1.57 (1.45–1.71)	< 0.001
**Livestock meat**	0.90 (0.71–1.18)	0.440	–	–	1.00 (0.88–1.14)	0.988	–	–	0.83 (0.76–0.89)	< 0.001	0.88 (0.81–0.95)	0.001
**Poultry meat**	0.77 (0.63–0.95)	0.014	0.89 (0.72–1.10)	0.290	0.66 (0.59–0.73)	< 0.001	0.73 (0.66–0.81)	< 0.001	0.59 (0.56–0.64)	< 0.001	0.62 (0.58–0.67)	< 0.001
**Seafood**	0.99 (0.77–1.27)	0.967	–	–	1.08 (0.95–1.21)	0.220	–	–	0.86 (0.80–0.94)	< 0.001	0.89 (0.82–0.97)	0.007
**Fruits**	0.78 (0.73–0.83)	< 0.001	0.84 (0.69–1.02)	0.085	0.79 (0.71–0.87)	< 0.001	0.89 (0.80–0.98)	0.015	0.70 (0.58–0.85)	< 0.001	0.83 (0.78–0.88)	< 0.001
**Spring onion** **/ginger/garlic**	0.78 (0.64–0.95)	0.014	0.85 (0.70–1.04)	0.116	0.88 (0.80–0.97)	0.009	0.93 (0.85–1.03)	0.188	0.68 (0.64–0.72)	< 0.001	0.69 (0.65–0.74)	< 0.001
**Nut**	0.93 (0.72–1.18)	0.537	–	–	0.83 (0.73–0.94)	0.003	0.89 (0.78–1.01)	0.067	0.74 (0.68–0.80)	< 0.001	0.77 (0.71–0.84)	< 0.001
**Milk**	0.99 (0.76–1.27)	0.937	–	–	0.81 (0.71–0.93)	0.003	0.82 (0.71–0.94)	0.004	0.99 (0.91–1.07)	0.828	–	–
**Vitamins**	0.72 (0.26–1.56)	0.464	–	–	0.43 (0.24–0.72)	0.003	0.49 (0.27–0.82)	0.012	0.64 (0.47–0.85)	0.003	0.68 (0.50–0.90)	0.010
**Leftovers**	1.46 (1.38–1.55)	< 0.001	1.16 (0.96–1.41)	0.125	1.07 (0.98–1.18)	0.150	1.08 (0.98–1.19)	0.108	1.16 (0.96–1.40)	0.119	1.49 (1.40–1.58)	< 0.001
**Physical exercise**	0.94 (0.71–1.23)	0.675	–	–	0.85 (0.74–0.98)	0.023	0.89 (0.77–1.03)	0.119	0.67 (0.61–0.74)	< 0.001	0.69 (0.62–0.76)	< 0.001
**Housework**												
No	1.00 (reference)	–	1.00 (reference)	–	1.00 (reference)	–	1.00 (reference)	–	1.00 (reference)	–	1.00 (reference)	–
< 8 h/week	0.97 (0.71–1.34)	0.859	1.11 (0.81–1.52)	0.531	0.86 (0.73–1.01)	0.057	0.92 (0.78–1.08)	0.317	0.59 (0.54–0.65)	< 0.001	0.61 (0.56–0.68)	< 0.001
8-14 h/week	0.78 (0.57–1.08)	0.123	0.97 (0.70–1.34)	0.845	0.79 (0.67–0.93)	0.004	0.88 (0.75–1.05)	0.147	0.54 (0.49–0.59)	< 0.001	0.57 (0.52–0.63)	< 0.001
15-21 h/week	0.80 (0.56–1.15)	0.217	0.95 (0.66–1.38)	0.796	0.78 (0.65–0.93)	0.006	0.84 (0.70–1.01)	0.066	0.56 (0.50–0.62)	< 0.001	0.58 (0.52–0.65)	< 0.001
≥ 22 h/week	0.65 (0.44–0.96)	0.029	0.79 (0.53–1.18)	0.249	0.98 (0.83–1.17)	0.843	1.06 (0.88–1.27)	0.542	0.61 (0.55–0.68)	< 0.001	0.63 (0.56–0.71)	< 0.001
**Snore**	1.11 (0.92–1.34)	0.273	–	–	1.07 (0.97–1.17)	0.172	1.04 (0.95–1.15)	0.414	1.11 (1.04–1.18)	0.001	1.09 (1.02–1.15)	0.009

Source: WHO tumor histological classification 2000

*BMI* body mass index (kg/m^2^), *LGIN* low-grade intraepithelial neoplasia, *HGIN* high-grade intraepithelial neoplasia, *ESCC* esophageal squamous cell carcinoma

^a^adjusted for age, gender, education, BMI and income

^b^Only part of the data with tea drinking temperature was analyzed, not all

**Table 4:** Medical history associated with different esophageal lesions

Medical history	HGIN/ESCC				LGIN				Esophagitis			
	OR (95% CI)	*P* value	Adjusted OR ^a^ (95% CI)	*P* value	OR (95% CI)	*P* value	Adjusted OR ^a^ (95% CI)	*P* value	OR (95% CI)	*P* value	Adjusted OR ^a^ (95% CI)	*P* value
**Take an acid suppressant**	0.65 (0.39–1.02)	0.077	0.68 (0.41–1.07)	0.119	0.49 (0.37–0.63)	< 0.001	0.51 (0.39–0.66)	< 0.001	0.68 (0.59–0.79)	< 0.001	0.70 (0.60–0.80)	< 0.001
**Take antibiotics**												
Not take	1.00 (reference)	–	1.00 (reference)	–	1.00 (reference)	–	1.00 (reference)	–	1.00 (reference)	–	1.00 (reference)	–
Not every week	0.27 (0.07–1.08)	0.063	0.34 (0.08–1.36)	0.127	0.76 (0.12–0.72)	0.180	0.90 (0.59–1.36)	0.617	0.54 (0.40–0.73)	< 0.001	0.61 (0.45–0.83)	0.001
Not every day	0.68 (0.17–2.74)	0.588	0.81 (0.20–3.28)	0.764	0.80 (0.43–1.51)	0.492	0.93 (0.49–1.77)	0.826	0.70 (0.461.08)	0.107	0.75 (0.49–1.16)	0.197
Every day	0.25 (0.04–1.80)	0.169	0.28 (0.04–1.99)	0.202	0.30 (0.12–0.72)	0.007	0.33 (0.14–0.80)	0.014	0.39 (0.24–0.63)	< 0.001	0.40 (0.24–0.65)	< 0.001
**Number of teeth lost**												
None	1.00 (reference)	–	1.00 (reference)	–	1.00 (reference)	–	1.00 (reference)	–	1.00 (reference)	–	1.00 (reference)	–
1–3	1.07 (0.84–1.36)	0.570	0.86 (0.68–1.09)	0.210	1.38 (1.24–1.54)	< 0.001	1.17 (1.04–1.31)	0.007	1.24 (1.16–1.33)	< 0.001	1.14 (1.06–1.23)	< 0.001
4–6	1.68 (1.26–2.22)	< 0.001	1.11 (0.83–1.48)	0.484	1.53 (1.31–1.77)	< 0.001	1.12 (0.96–1.30)	0.137	1.35 (1.22–1.48)	< 0.001	1.15 (1.04–1.27)	0.005
7–11	1.54 (1.01–2.26)	0.035	0.83 (0.55–1.25)	0.382	1.88 (1.55–2.26)	< 0.001	1.19 (0.98–1.44)	0.077	1.61 (1.41–1.82)	< 0.001	1.27 (1.12–1.45)	< 0.001
12–31	2.62 (1.70–3.87)	< 0.001	1.21 (0.79–1.84)	0.385	2.18 (1.73–2.72)	< 0.001	1.26 (0.99–1.58)	0.054	2.05 (1.76–2.37)	< 0.001	1.54 (1.32–1.79)	< 0.001
Complete denture	2.72 (1.72–4.10)	< 0.001	1.20 (0.77–1.87)	0.420	2.55 (2.02–3.19)	< 0.001	1.42 (1.11–1.78)	0.004	2.66 (2.30–3.07)	< 0.001	1.94 (1.66–2.25)	< 0.001
**Loose teeth**	1.89 (1.27–2.70)	0.001	1.48 (0.99–2.12)	0.042	1.49 (1.21–1.82)	< 0.001	1.23 (1.00–1.51)	0.049	1.23 (1.06–1.42)	0.005	1.10 (0.95–1.27)	0.202

Source: WHO tumor histological classification 2000

*BMI* body mass index (kg/m^2^), *LGIN* low-grade intraepithelial neoplasia, *HGIN* high-grade intraepithelial neoplasia, *ESCC* esophageal squamous cell carcinoma

^a^adjusted for age, gender, education, BMI and income

After final unconditional multivariable analysis, we identified a few important risk factors of 3 esophageal lesions. For the HGIN/ESCC patients, some unhealthy lifestyle and dietary habits (Table 3) were risk factors, including alcohol (adjusted OR 1.60, 95%CI 1.23–2.09), smoking (adjusted OR 1.46, 95%CI 1.14–1.89), drinking well and surface water (adjusted OR 1.55, 95%CI 1.28–1.89), salty diet (adjusted OR 1.36, 95%CI 1.07–1.77), drinking tea (adjusted OR 1.32, 95%CI 1.08–1.61). Although the *P* value of drinking hot/burning hot tea was no statistically significant, the adjusted OR value was 1.34. In addition, pesticide exposure (adjusted OR 1.44, 95% CI 1.11–1.84), loose teeth (adjusted OR 1.48, 95%CI 0.99–2.12), history of chronic hepatitis/cirrhosis (adjusted OR 1.91, 95%CI 1.03–3.22), and family history of cancer (adjusted OR 1.64, 95%CI 1.34–2.00), also positively related to the risk of HGIN/ESCC (Tables 2, 3, 4).

Based on the results of the adjusted analysis, we also found that some risk factors associated with LGIN were the same as HGIN/ESCC, consisting of drinking alcohol (adjusted OR 1.30, 95%CI 1.12–1.51), drinking well and surface water (adjusted OR 1.19, 95%CI 1.08–1.32), salty diet (adjusted OR 1.57, 95%CI 1.38–1.81), drinking tea (adjusted OR 1.34, 95%CI 1.26–1.54), family history of cancer (adjusted OR 1.33, 95%CI 1.20–1.47), pesticide exposure (adjusted OR 1.20, 95%CI 1.05–1.37) and loose teeth (adjusted OR 1.23, 95%CI 1.00–1.51). In addition, occupation as a farmer (adjusted OR 1.20, 95%CI 1.07–1.35) was also positively related to the risk of LGIN (Table 3). Drinking hot tea still did not find statistical significance (adjusted OR 1.10, 95%CI 0.92–1.33). On the contrary, eating poultry meat (adjusted OR 0.73, 95%CI 0.66–0.81), fruits (adjusted OR 0.89, 95%CI 0.80–0.98), vitamins (adjusted OR 0.49, 95%CI 0.27–0.82), milk (adjusted OR 0.82, 95%CI 0.71–0.94), acid suppressants (adjusted OR 0.51, 95%CI 0.39–0.66), antibiotics (adjusted OR 0.33, 95%CI 0.14–0.80) and drinking improved water (adjusted OR 0.58, 95%CI 0.46–0.72) were negatively related to the risk of LGIN.

Although esophagitis is a benign disease, there are more risk elevating and risk reduction factors than the diseases mentioned above. For the diet habits (Table 3), except that leftovers (adjusted OR 1.49, 95%CI 1.40–1.58) and salty diet (adjusted OR 1.57, 95%CI 1.45–1.71) were risk factors, the remaining factors had protective effect on esophagitis, such as poultry meat (adjusted OR 0.62, 95%CI 0.58–0.67), fruits (adjusted OR 0.83, 95%CI 0.78–0.88), vitamins (adjusted OR 0.68, 95%CI 0.50–0.90), livestock meat (adjusted OR 0.88, 95%CI 0.81–0.95), seafood (adjusted OR 0.89, 95%CI 0.82–0.97), spring onion/ginger/garlic (adjusted OR 0.69, 95%CI 0.65–0.74) and nuts (adjusted OR 0.77, 95%CI 0.71–0.84).

In addition, some unhealthy lifestyles were positively related to the risk of esophagitis (Table 3), including smoking (adjusted OR 1.09, 95%CI 1.00–1.19), drinking well and surface water (adjusted OR 1.61, 95%CI 1.51–1.71), and having snore (adjusted OR 1.09, 95%CI 1.02–1.15). On the contrary, other healthy lifestyles were negatively related to the risk of esophagitis (Table 3), including drinking improved water (adjusted OR 0.73, 95%CI 0.64–0.83), refrigerator use (adjusted OR 0.50–0.73, *P* < 0.001), physical exercise (adjusted OR 0.69, 95%CI 0.62–0.76) and doing housework (adjusted OR 0.57–0.63, *P* < 0.001). Except for foods and lifestyles, family history of cancer (adjusted OR 1.22, 95%CI 1.14–1.30), history of chronic hepatitis/cirrhosis (adjusted OR 2.96, 95%CI 2.52–3.47), occupation as a farmer (adjusted OR 1.21, 95%CI 1.12–1.30) and missing teeth (adjusted OR 1.14–1.94, *P* < 0.01) were positively related to the risk of esophagitis (Tables 2, 3, 4). Taking acid suppressants (adjusted OR 0.70, 95%CI 0.60–0.80) and taking antibiotics every day (adjusted OR 0.40, 95%CI 0.24–0.65) were negatively related to the risk of esophagitis (Table 4).

## Discussion

The high rate of early esophageal lesions progressing to esophageal cancer has resulted in a concerted attempt to better understand the risk factors associated with this difference. In this study, we conducted a cross-sectional study in five areas with high incidence of ESCC in China, aiming to recognize the risk factors related to different esophageal lesions. At last, we identified three important risk factors, which persisted across all stages. Otherwise, we also find that the key risk factors in different stages were various.

The three common risk factors are drinking well and surface water, salty diet, and family history of cancer. At present, the main sources of drinking water for some local residents in rural area of high incidence region were still well and surface water. Since the 1980s, the local government has taken significant measures to improve the quality of drinking water, and the incidence and mortality of esophageal cancer displayed a decreasing trend compared with regions where no improvement [23]. Our result showed that drinking well and surface water was important risk factor from early stage to late stage of lesions. We advised that the water improvement project still needed to implement in rural regions with poor drinking water quality.

Previous studies found local people of rural areas were inclined to eat salty foods, such as salted meat, salted duck eggs and pickled vegetables. Both high frequency and long-term intakes were associated with elevated risk of ESCC. This observation is consistent with other studies [16, 24, 25]. However, the underlying mechanism was still unclear. We speculate that the mechanism, possible similar to gastric cancer [26, 27], is that high salt concentrations disrupt the mucosal barrier and lead to inflammation, which could increase the possibility of bacterial infection. It is noticed that salty diet, an important risk factor, existed at all stages of early esophageal lesions in our study.

The association between ESCC and family history of cancer had been reported. Some studies showed that family history of cancer was strongly associated with ESCC risk and that genetic factors could contribute to the effect [28, 29]. Based on our results, we suggest that the above three common risk factors should be considered first in prevention of all stages.

History of chronic hepatitis/cirrhosis, pesticides exposure and alcohol were important risk factors for esophagitis, LGIN and HGIN/ESCC, respectively. China has a high burden of liver disease [30, 31], previous studies report that chronic liver diseases, in particular chronic hepatitis B virus infection, are associated with reflux esophagitis reflux [32, 33]. In this study, we identified that history of chronic hepatitis/cirrhosis was the tremendous risk factor both esophagitis and HGIN/ESCC. Currently, there is an effective vaccine to prevent hepatitis B virus infection. Increased vaccination for hepatitis B could also reduce the occurrence of ESCC in high incidence regions. Similar to our findings, previous studies had confirmed that alcohol is a risk factor for ESCC [4, 10]. The reason is that acetaldehyde, a class 1 carcinogen, is the first metabolite of ethanol metabolism [34, 35].

Pesticides had been reported as a risk factor for a variety of cancers [36], such as prostate cancer [37] and childhood leukemia [38], but the role of pesticides in esophageal cancer is still unclear. Our results showed that pesticide exposure was an important risk factor, which not only increased the risk of LGIN (adjusted OR 1.20, 95%CI 1.05–1.37) but also HGIN/ESCC (adjusted OR 1.44, 95%CI 1.11–1.84). In this study, most of participants are farmer, who had a greater chance exposed pesticide than non-farmer, which could increase the risk of early esophageal lesions. Therefore, the result showed that pesticide was a significant risk factor of LGIN and HGIN/ESCC. Further study is needed to evaluate the mechanism linking pesticide exposure and early esophageal lesions.

It is noticed that our study found that high frequency of drinking tea is a risk factor for LGIN and LGIN/ESCC, which is similar to previous study [39]. Currently, the association between tea consumption and esophageal cancer is inconsistent, which could be related to the type of tea [40]. In addition, our research found that although adjusted OR values were all greater than 1, there was no statistically significant correlation between high tea temperature and different esophageal lesions. This result could be caused by the small sample size of cases. Further prospective study is still needed. Interestingly, our results revealed that antibiotics were protective factor for esophagitis and LGIN. Previous study found that *Porphyromonas gingivalis* was positively correlated with the severity of esophageal squamous cell carcinoma and might contribute to the pathogenesis of esophageal squamous cell carcinoma [41]. We speculated that antibiotics intake might change the composition of the gut microbiome and reduce their adverse effects on esophagus, thus play a positive role in the prevention of ESCC. Besides, we also found that with the aggravation of the esophageal lesions, the number of risk factors reduced, which suggested that the effect of environmental factors on the late stage of the disease decreased. These results also mean that it is a more meaningful method for prevention of ESCC by controlling environmental factors in early lesion, such as balanced diet, proper exercise, and healthy lifestyle.

The present study had several limitations. Most of the variables analyzed in the study were based on self-reported data which may be susceptible to recall bias. In addition, there are some factors were not collected in the questionnaire, such as cooking methods, history of upper gastrointestinal tumors or esophageal cancer. The main strength of our study is that it was the first multicenter cross-sectional study to identify risk factors in different stage of early esophageal lesions. Furthermore, detailed questionnaire was collected in a standardized data entry system by trained investigator to assure the quality of the data. In addition, we used the gold standard method to exam esophageal lesions.

## Conclusions

In conclusion, we performed a cross sectional study of 44,857 individuals in the age range of 40–69 years. These individuals live in regions with high-rates of ESCC regions in China. This report has identified drinking well and surface water, salty diet, family history of cancer and alcohol consumption as factors influencing disease rates across all stages. We also identified history of chronic hepatitis/cirrhosis and pesticide exposure as essential risk factors in esophagitis, LGIN and HGIN/ESCC, respectively. The results make a compelling case for environmental factors being the main driver of disease. The results also suggest directions for efforts to ESCC in an early stage of disease in high incidence regions. However, considering the complexity of the disease, although environmental factors play an important role in the early stages of ESCC, these may vary as the severity of the disease increases. Further cohort studies are needed to support and expand our understanding of ESCC risk factors.

## Supplementary Information

**Additional file 1.**

**Additional file 2.**

**Additional file 3.**

**Additional file 4.**

**Additional file 5.**

## Data Availability

The datasets used and/or analysed during the current study are available from the corresponding author on reasonable request.

## Abbreviations

BMI: Body mass index
CIs: Confidence intervals
CIS: Carcinoma in situ
EADC: Esophageal adenocarcinoma
ESCC: Esophageal squamous cell carcinoma
ESD: Esophageal squamous dysplasia
ESSH: Esophageal squamous simple hyperplasia
HGIN: High-grade intraepithelial neoplasia
LGIN: Low-grade intraepithelial neoplasia
ORs: Odds ratios
