# Supplementary material for: Risk factors for esophageal squamous cell carcinoma and its histological precursor lesions in China: a multicenter cross-sectional study
Source: BMC Cancer. 2021 Sep 16;21:1034. doi: 10.1186/s12885-021-08764-x (PMC8444572; doi:10.1186/s12885-021-08764-x)
Supplement: Supplementary file 2 — Additional file 2. [file 12885_2021_8764_MOESM2_ESM.doc]

***Cigarette Smoking and Alcohol Drinking***

Participants were asked about their lifetime smoking and alcohol drinking history. Cigarette smoking was defined as a smoking history of at least one or more cigarette a day for half year currently or former, and alcohol drinking was defined as drinking alcohol at least one day a week for 12 months currently or former. NO means not smoking or drinking, and YES means former/current smoking or drinking.

***Tea Drinking***

Tea drinking was defined as a drinking history of at least one day a week currently or former. NO means not drinking, and YES means former/current drinking.

***Tea Temperature***

In our study, participants who reported weekly consumption were asked the usual temperature of the tea (room temperature or warm, hot, or burning hot).

***Cancer Family History***

The type of cancer family history included all cancers. Cancer family history was defined as the direct blood relatives and collateral blood relatives within 3 generations. Relatives included people who are still alive or died. All cancers should be based on the formal diagnosis of doctors at township/district level hospitals or above, rather than personal inferences.

***Diet Taste***

This question had 2 options: (a) salty diet, and (b) light diet. Salty diet was defined as consuming at least 320g salt a month. Light diet was defined as consuming less than 320 g salt a month.

***Pesticide Exposure***

This question had 2 options: (a) no and (b) yes.

***Source of Drinking Water***

This question had 5 options: (a) cellar water, pond water, and shallow well water, (b) lake water and river water, (c) deep well water and spring water, (d) Tap water, (e) pure water. Subjects selecting (d) and (e) were classified as tap/pure water, and the others were classified as well water and surface water.

***Drink Improved Water***

This question had 2 options: (a) no and (b) yes.

***Dietary Habits***

***Livestock meat***. This question had 5 options: (a) don't eat / eat very little, (b) 1-3 days/month, (c) 1-3 days/week, (d) 4-6 days/week, and every day. Subjects selecting (a) were classified as not eating, and those selecting others as eating.

***Poultry meat***. This question had 5 options: (a) don't eat / eat very little, (b) 1-3 days/month, (c) 1-3 days/week, (d) 4-6 days/week, and every day. Subjects selecting (a) were classified as not eating, and those selecting others as eating.

***Seafood***. This question had 5 options: (a) don't eat / eat very little, (b) 1-3 days/month, (c) 1-3 days/week, (d) 4-6 days/week, and every day. Subjects selecting (a) were classified as not eating, and those selecting others as eating.

***Fruits***. This question had 5 options: (a) don't eat / eat very little, (b) 1-3 days/month, (c) 1-3 days/week, (d) 4-6 days/week, and every day. Subjects selecting (a) were classified as not eating, and those selecting others as eating.

***Bean productss***. This question had 5 options: (a) don't eat / eat very little, (b) 1-3 days/month, (c) 1-3 days/week, (d) 4-6 days/week, and every day. Subjects selecting (a) were classified as not eating, and those selecting others as eating.

***Spring onion/ginger/garlic***. This question had 5 options: (a) don't eat / eat very little, (b) 1-3 days/month, (c) 1-3 days/week, (d) 4-6 days/week, and every day. Subjects selecting (a) were classified as not eating, and those selecting others as eating.

***Nut***. This question had 5 options: (a) don't eat / eat very little, (b) 1-3 days/month, (c) 1-3 days/week, (d) 4-6 days/week, and every day. Subjects selecting (a) were classified as not eating, and those selecting others as eating.

***Milk***. This question had 5 options: (a) don't eat / eat very little, (b) 1-3 days/month, (c) 1-3 days/week, (d) 4-6 days/week, and every day. Subjects selecting (a) were classified as not eating, and those selecting others as eating.

***Soybean milk***. This question had 5 options: (a) don't eat / eat very little, (b) 1-3 days/month, (c) 1-3 days/week, (d) 4-6 days/week, and every day. Subjects selecting (a) were classified as not eating, and those selecting others as eating.

***Vitamins***. This question had 5 options: (a) don't eat / eat very little, (b) 1-3 days/month, (c) 1-3 days/week, (d) 4-6 days/week, and every day. Subjects selecting (a) were classified as not eating, and those selecting others as eating.

***Leftovers***. This question had 5 options: (a) don't eat / eat very little, (b) 1-3 days/month, (c) 1-3 days/week, (d) 4-6 days/week, and every day. Subjects selecting (a) were classified as not eating, and those selecting others as eating.

***Eat out***. This question had 5 options: (a) don't eat / eat very little, (b) 1-3 days/month, (c) 1-3 days/week, (d) 4-6 days/week, and every day. Subjects selecting (a) were classified as not eating, and those selecting others as eating.

***Cooking***

This question had 5 options: (a) cooking every day/almost every day, (b) cooking several times a week, (c) cooking several times a month, (d) not cooking / almost not cooking, and (e) No kitchen. Subjects selecting (d) and (e) were classified as not cooking, and those selecting others as cooking.

***Physical Exercise***

This question had 5 options: (a) never or almost not exercise, (b) 1-3 times a month, (c) 1-2 times a week, (d) 3-5 times a week, and (e) exercise every day or almost every day. Subjects selecting (a) were classified as not exercise, and those selecting others as exercise.

***Snore***

This question had 3 options: (a) always, (b) sometimes, and (c) no snoring. Subjects selecting (c) were classified as no snoring, and those selecting others as snoring.

***Nap***

This question had 4 options: (a) all seasons, (b) usually only in summer, (c) Other time (except the first two cases), and (d) no. Subjects selecting (d) were classified as not nap, and those selecting others as nap.

***Loose Teeth***

Loose teeth was defined as the vertical movement of the teeth exceeds 0.02 mm, which means that have the feeling of loose teeth. This question had 2 options: (a) no and (b) yes.

***History of Chronic Hepatitis and Cirrhosis***

The types of chronic hepatitis/cirrhosis included chronic hepatitis B, chronic hepatitis C, chronic alcoholic hepatitis, autoimmune liver disease, and cirrhosis. This question had 2 options: (a) no and (b) yes.

***Take an Acid Suppressant***

Acid suppressing drugs included proton pump inhibitor (PPI), H2 receptor inhibitor (H2RA), and anticholinergic and gastrin receptor antagonist. This question had 2 options: (a) no and (b) yes.

***Take Antibiotics***

Antibiotic drugs included penicillins, cephalosporins, aminoglycosides, macrolides, tetracyclines, chloramphenicol, lincosamides, peptides and other antibiotics, sulfonamides, quinolones and synthetic antibacterial drugs. This question had 4 options: (a) not take, (b) not every week, (c) not every day, and (d) every day.
