# Supplementary material for: Risk factors for esophageal squamous cell carcinoma and its histological precursor lesions in China: a multicenter cross-sectional study
Source: BMC Cancer. 2021 Sep 16;21:1034. doi: 10.1186/s12885-021-08764-x (PMC8444572; doi:10.1186/s12885-021-08764-x)
Supplement: Supplementary file 3 — Additional file 3. [file 12885_2021_8764_MOESM3_ESM.doc]

Supplementary table 1 Distribution of characteristics of different esophageal lesions

| **Factors** | **HGIN/ESCC**  **n = 437** | **LGIN**  **n = 1,874** | **Esophagitis**  **n = 4,890** | **Normal esophagus**  **n = 37,656** | **Total cohort**  **n = 44,857** | ***P* value** |
| --- | --- | --- | --- | --- | --- | --- |
| **Gender, n(%)** |  |  |  |  |  | <0.001^a^ |
| Male | 247 (56.5) | 913 (48.7) | 2290 (46.8) | 15590 (41.4) | 19040 (42.4) |  |
| Female | 190 (43.5) | 961 (51.3) | 2600 (53.2) | 22066 (58.6) | 25817 (57.6) |  |
| **Age, mean (SD)** | 60.85 (5.80) | 59.22 (6.25) | 57.29 (6.85) | 54.41 (7.26) | 54.99 (7.30) | <0.001^b^ |
| **Education, n(%)** |  |  |  |  |  | <0.001^a^ |
| No formal | 81 (18.5) | 368 (19.6) | 744 (15.2) | 5395 (14.3) | 6588 (14.7) |  |
| Formal | 356 (81.5) | 1506 (80.4) | 4146 (84.8) | 32261 (85.7) | 38269 (85.3) |  |
| **Occupation, n(%)** |  |  |  |  |  | <0.001^a^ |
| Non-farmer | 86 (19.7) | 382 (20.4) | 1028 (21.0) | 9347 (24.8) | 10843 (24.2) |  |
| Farmer | 351 (80.3) | 1492 (79.6) | 3862 (79.0) | 28309 (75.2) | 34014 (75.8) |  |
| **BMI, n(%)** |  |  |  |  |  | <0.001^a^ |
| < 18.5 | 16 (3.7) | 36 (1.9) | 98 (2.0) | 714 (1.9) | 864 (1.9) |  |
| 18.5 to 23.9 | 229 (52.3) | 914 (48.8) | 2336 (47.8) | 17309 (46.0) | 20788 (46.3) |  |
| 24.0 to 27.9 | 159 (36.4) | 742 (39.6) | 1977 (40.4) | 15074 (40.0) | 17952 (40.1) |  |
| ≥ 28.0 | 33 (7.6) | 182 (9.7) | 479 (9.8) | 4559 (12.1) | 5253 (11.7) |  |
| **Marital status, n(%)** |  |  |  |  |  | <0.001^a^ |
| Married | 401 (91.8) | 1757 (93.8) | 4659 (95.3) | 36139 (96.0) | 42956 (95.8) |  |
| Live alone | 36 (8.2) | 117 (6.2) | 231 (4.7) | 1517 (4.0) | 1901 (4.2) |  |
| **Pesticide exposure, n(%)** |  |  |  |  |  | <0.001^a^ |
| No | 362 (82.8) | 1596 (85.2) | 4264 (87.2) | 32983 (87.6) | 39205 (87.4) |  |
| Yes | 75 (17.2) | 278 (14.8) | 626 (12.8) | 4673 (12.4) | 5652 (12.6) |  |
| **Annual income per family (RMB), n(%)** |  |  |  |  |  | <0.001^a^ |
| <10,000 | 70 (16.0) | 227 (12.1) | 594 (12.1) | 4283 (11.3) | 5174 (11.5) |  |
| 10,000- | 117 (26.8) | 581 (31.0) | 1310 (26.8) | 10933 (29.0) | 12941 (28.8) |  |
| 30,000- | 162 (37.1) | 674 (36.0) | 1755 (35.9) | 12370 (32.9) | 14961 (33.5) |  |
| 50,000- | 63 (14.4) | 279 (14.9) | 773 (15.8) | 6395 (17.0) | 7510 (16.7) |  |
| ≥70,000 | 25 (5.7) | 113 (6.0) | 458 (9.4) | 3675 (9.8) | 4271 (9.5) |  |
| **Smoking, n(%)** |  |  |  |  |  | <0.001^a^ |
| Not smoke | 284 (65.0) | 1388 (74.1) | 3649 (74.6) | 29578 (78.5) | 34899 (77.8) |  |
| Former/current smoke | 153 (35.0) | 486 (25.9) | 1241 (25.4) | 8078 (21.5) | 9958 (22.2) |  |
| **Alcohol, n(%)** |  |  |  |  |  | <0.001^a^ |
| Not drink | 358 (81.9) | 1619 (86.4) | 4348 (88.9) | 34152 (90.7) | 40477 (90.2) |  |
| Former/current drink | 79 (18.1) | 255 (13.6) | 542 (11.1) | 3504 (9.3) | 4380 (9.8) |  |
|  |  |  |  |  |  |  |
|  |  |  |  |  |  |  |
|  |  |  |  |  |  |  |
| Supplementary table 1 Distribution of characteristics of different esophageal lesions (continued) | | | | | | |
| **Factors** | **HGIN/ESCC**  **n = 437** | **LGIN**  **n = 1,874** | **Esophagitis**  **n = 4,890** | **Normal esophagus**  **n = 37,656** | **Total cohort**  **n = 44,857** | ***P* value** |
| **Tea drinking frequency, n(%)** |  |  |  |  |  | <0.001^a^ |
| Not drink | 286 (65.4) | 1203 (64.2) | 3753 (76.7) | 27077 (71.9) | 32319 (72.0) |  |
| Former/current drink | 151 (34.6) | 671 (35.8) | 1137 (23.3) | 10579 (28.1) | 12538 (28.0) |  |
| **Tea temperature ^c^, n(%)** |  |  |  |  |  | 0.206 |
| Warm | 32 (21.3) | 165 (24.7) | 280 (24.9) | 2769 (26.7) | 3246 (26.3) |  |
| Hot/burning hot tea | 118 (78.7) | 502 (75.3) | 845 (75.1) | 7619 (73.3) | 9084 (73.7) |  |
| **Source of drinking water, n(%)** |  |  |  |  |  | <0.001^a^ |
| Tap/pure water | 226 (51.7) | 1095 (58.4) | 2563 (52.4) | 23980 (63.7) | 27864 (62.1) |  |
| Well water and surface water | 211 (48.3) | 779 (41.6) | 2327 (47.6) | 13676 (36.3) | 16993 (37.9) |  |
| **Drink improved water, n(%)** |  |  |  |  |  | <0.001^a^ |
| No | 410 (93.8) | 1786 (95.3) | 4596 (94.0) | 34591 (91.9) | 41383 (92.3) |  |
| Yes | 27 (6.2) | 88 (4.7) | 294 (6.0) | 3065 (8.1) | 3474 (7.7) |  |
| **Livestock meat, n(%)** |  |  |  |  |  | <0.001^a^ |
| No | 72 (16.5) | 284 (15.2) | 868 (17.8) | 5702 (15.1) | 6926 (15.4) |  |
| Yes | 365 (83.5) | 1590 (84.8) | 4022 (82.2) | 31954 (84.9) | 37931 (84.6) |  |
| **Poultry meat, n(%)** |  |  |  |  |  | <0.001^a^ |
| No | 301 (68.9) | 1353 (72.2) | 3625 (74.1) | 23781 (63.2) | 29060 (64.8) |  |
| Yes | 136 (31.1) | 521 (27.8) | 1265 (25.9) | 13875 (36.8) | 15797 (35.2) |  |
| **Seafood, n(%)** |  |  |  |  |  | 0.002^a^ |
| No | 361 (82.6) | 1526 (81.4) | 4134 (84.5) | 31079 (82.5) | 37100 (82.7) |  |
| Yes | 76 (17.4) | 348 (18.6) | 756 (15.5) | 6577 (17.5) | 7757 (17.3) |  |
| **Fruits, n(%)** |  |  |  |  |  | <0.001^a^ |
| No | 182 (41.6) | 731 (39.0) | 1918 (39.2) | 12599 (33.5) | 15430 (34.4) |  |
| Yes | 255 (58.4) | 1143 (61.0) | 2972 (60.8) | 25057 (66.5) | 29427 (65.6) |  |
| **Bean products, n(%)** |  |  |  |  |  | 0.253^a^ |
| No | 216 (49.4) | 937 (50.0) | 2422 (49.5) | 19176 (50.9) | 22751 (50.7) |  |
| Yes | 221 (50.6) | 937 (50.0) | 2468 (50.5) | 18480 (49.1) | 22106 (49.3) |  |
| **Spring onion/ginger/garlic, n(%)** |  |  |  |  |  | <0.001^a^ |
| No | 161 (36.8) | 641 (34.2) | 1965 (40.2) | 11799 (31.3) | 14566 (32.5) |  |
| Yes | 276 (63.2) | 1233 (65.8) | 2925 (59.8) | 25857 (68.7) | 30291 (67.5) |  |
|  |  |  |  |  |  |  |
| Supplementary table 1 Distribution of characteristics of different esophageal lesions (continued) | | | | | | |
| **Factors** | **HGIN/ESCC**  **n = 437** | **LGIN**  **n = 1,874** | **Esophagitis**  **n = 4,890** | **Normal esophagus**  **n = 37,656** | **Total cohort**  **n = 44,857** | ***P* value** |
| **Nut, n(%)** |  |  |  |  |  | <0.001^a^ |
| No | 359 (82.2) | 1569 (83.7) | 4168 (85.2) | 30496 (81.0) | 36592 (81.6) |  |
| Yes | 78 (17.8) | 305 (16.3) | 722 (14.8) | 7160 (19.0) | 8265 (18.4) |  |
| **Milk, n(%)** |  |  |  |  |  | 0.029^a^ |
| No | 368 (84.2) | 1624 (86.7) | 4117 (84.2) | 31658 (84.1) | 37767 (84.2) |  |
| Yes | 69 (15.8) | 250 (13.3) | 773 (15.8) | 5998 (15.9) | 7090 (15.8) |  |
| **Soybean milk, n(%)** |  |  |  |  |  | 0.002^a^ |
| No | 422 (96.6) | 1810 (96.6) | 4702 (96.2) | 35879 (95.3) | 42813 (95.4) |  |
| Yes | 15 (3.4) | 64 (3.4) | 188 (3.8) | 1777 (4.7) | 2044 (4.6) |  |
| **Vitamins, n(%)** |  |  |  |  |  | <0.001^a^ |
| No | 432 (98.9) | 1861 (99.3) | 4840 (99.0) | 37059 (98.4) | 44192 (98.5) |  |
| Yes | 5 (1.1) | 13 (0.7) | 50 (1.0) | 597 (1.6) | 665 (1.5) |  |
| **Leftovers, n(%)** |  |  |  |  |  | <0.001^a^ |
| No | 232 (53.1) | 1033 (55.1) | 2316 (47.4) | 21393 (56.8) | 24974 (55.7) |  |
| Yes | 205 (46.9) | 841 (44.9) | 2574 (52.6) | 16263 (43.2) | 19883 (44.3) |  |
| **Eat out, n(%)** |  |  |  |  |  | 0.004^a^ |
| No | 387 (88.6) | 1699 (90.7) | 4314 (88.2) | 33096 (87.9) | 39496 (88.0) |  |
| Yes | 50 (11.4) | 175 (9.3) | 576 (11.8) | 4560 (12.1) | 5361 (12.0) |  |
| **Diet taste, n(%)** |  |  |  |  |  | <0.001^a^ |
| Light diet | 74 (16.9) | 283 (15.1) | 740 (15.1) | 8349 (22.2) | 9446 (21.1) |  |
| Salty diet | 363 (83.1) | 1591 (84.9) | 4150 (84.9) | 29307 (77.8) | 35411 (78.9) |  |
| **Refrigerator, n(%)** |  |  |  |  |  | <0.001^a^ |
| No refrigerator | 52 (11.9) | 179 (9.6) | 508 (10.4) | 2655 (7.1) | 3394 (7.6) |  |
| 1-10years | 324 (74.1) | 1437 (76.7) | 3682 (75.3) | 28965 (76.9) | 34408 (76.7) |  |
| 11-20years | 58 (13.3) | 244 (13.0) | 668 (13.7) | 5689 (15.1) | 6659 (14.8) |  |
| >20years | 3 (0.7) | 14 (0.7) | 32 (0.7) | 347 (0.9) | 396 (0.9) |  |
| **Cooking, n(%)** |  |  |  |  |  | <0.001^a^ |
| No | 149 (34.1） | 617 (32.9) | 1573 (32.2) | 10566 (28.1) | 12905 (28.8) |  |
| Yes | 288 (65.9) | 1257 (67.1) | 3317 (67.8) | 27090 (71.9) | 31952 (71.2) |  |
| **Physical exercise, n(%)** |  |  |  |  |  | <0.001^a^ |
| No | 378 (86.5) | 1643 (87.7) | 4402 (90.0) | 32307 (85.8) | 38730 (86.3) |  |
| Yes | 59 (13.5) | 231 (12.3) | 488 (10.0) | 5349 (14.2) | 6127 (13.7) |  |
| **Snore, n(%)** |  |  |  |  |  | 0.004^a^ |
| No | 247 (56.5) | 1078 (57.5) | 2767 (56.6) | 22260 (59.1) | 26352 (58.7) |  |
| Yes | 190 (43.5) | 796 (42.5) | 2123 (43.4) | 15396 (40.9) | 18505 (41.3) |  |
|  |  |  |  |  |  |  |
|  |  |  |  |  |  |  |
| Supplementary table 1 Distribution of characteristics of different esophageal lesions (continued) | | | | | | |
| **Factors** | **HGIN/ESCC**  **n = 437** | **LGIN**  **n = 1,874** | **Esophagitis**  **n = 4,890** | **Normal esophagus**  **n = 37,656** | **Total cohort**  **n = 44,857** | ***P* value** |
| **Housework, n(%)** |  |  |  |  |  | <0.001^a^ |
| No | 54 (12.4) | 227 (12.1) | 826 (16.9) | 3904 (10.4) | 5011 (11.1) |  |
| <8h/week | 142 (32.5) | 526 (28.1) | 1317 (26.9) | 10564 (28.0) | 12549 (28.0) |  |
| 8-14h/week | 122 (27.9) | 521 (27.8) | 1290 (26.4) | 11369 (30.2) | 13302 (29.7) |  |
| 15-21h/week | 69 (15.8) | 282 (15.0) | 737 (15.1) | 6252 (16.6) | 7340 (16.4) |  |
| ≥22h/week | 50 (11.4) | 318 (17.0) | 720 (14.7) | 5567 (14.8) | 6655 (14.8) |  |
| **Nap, n(%)** |  |  |  |  |  | 0.006^a^ |
| No | 156 (35.7) | 652 (34.8) | 1555 (31.8) | 11850 (31.5) | 14213 (31.7) |  |
| Yes | 281 (64.3) | 1222 (65.2) | 3335 (68.2) | 25806 (68.5) | 30644 (68.3) |  |
| **Number of teeth lost, n(%)** |  |  |  |  |  | <0.001^a^ |
| Never | 176 (40.3) | 711 (37.9) | 1972 (40.3) | 18402 (48.9) | 21261 (47.4) |  |
| 1-3 | 114 (26.0) | 594 (31.7) | 1481 (30.3) | 11128 (29.6) | 13317 (29.7) |  |
| 4-6 | 68 (15.6) | 249 (13.3) | 610 (12.5) | 4224 (11.2) | 5151 (11.5) |  |
| 7-11 | 28 (6.4) | 138 (7.3) | 327 (6.7) | 1900 (5.0) | 2393 (5.3) |  |
| 12-31 | 27 (6.2) | 91 (4.9) | 237 (4.8) | 1079 (2.9) | 1434 (3.2) |  |
| Complete denture | 24 (5.5) | 91 (4.9) | 263 (5.4) | 923 (2.4) | 1301 (2.9) |  |
| **Loose teeth, n(%)** |  |  |  |  |  | <0.001^a^ |
| No | 407 (93.1) | 1771 (94.5) | 4666 (95.4) | 36241 (96.2) | 43085 (96.0) |  |
| Yes | 30 (6.9) | 103 (5.5) | 224 (4.6) | 1415 (3.8) | 1772 (4.0) |  |
| **History of chronic hepatitis and cirrhosis, n(%)** |  |  |  |  |  | <0.001^a^ |
| No | 424 (97.0) | 1835 (97.9) | 4667 (95.4) | 37076 (98.5) | 44002 (98.1) |  |
| Yes | 13 (3.0) | 39 (2.1) | 223 (4.6) | 580 (1.5) | 855 (1.9) |  |
| **Family history of cancer, n(%)** |  |  |  |  |  | <0.001^a^ |
| No | 281 (64.3) | 1283 (68.5) | 3395 (69.4) | 27460 (72.9) | 32419 (72.3) |  |
| Yes | 156 (35.7) | 591 (31.5) | 1495 (30.6) | 10196 (27.1) | 12438 (27.7) |  |
| **Take an acid suppressant, n(%)** |  |  |  |  |  | <0.001^a^ |
| No | 419 (95.9) | 1816 (96.9) | 4680 (95.7) | 35330 (93.8) | 42245 (94.2) |  |
| Yes | 18 (4.1) | 58 (3.1) | 210 (4.3) | 2326 (6.2) | 2612 (5.8) |  |
| **Take antibiotics, n(%)** |  |  |  |  |  | <0.001^a^ |
| Not take | 432 (98.8) | 1835 (97.9) | 4805 (98.3) | 36442 (96.7) | 43514 (97.0) |  |
| Not every week | 2 (0.5) | 24 (1.3) | 45 (0.9) | 631 (1.7) | 358 (0.8) |  |
| Not every day | 2 (0.5) | 10 (0.5) | 23 (0.5) | 248 (0.7) | 283 (0.6) |  |
| Every day | 1 (0.2) | 5 (0.3) | 17 (0.3) | 335 (0.9) | 702 (1.6) |  |

**Legend:** BMI = body mass index (kg/m^2^). LGIN = low-grade intraepithelial neoplasia; HGIN = high-grade intraepithelial neoplasia; ESCC = esophageal squamous cell carcinoma. Source: WHO tumor histological classification. ^a^ = *χ^2^* test; ^b^ = *ANOVA* test; ^c^ = Only part of the data with tea drinking temperature was analyzed, not all.
