# Supplementary material for: Risk factors for esophageal squamous cell carcinoma and its histological precursor lesions in China: a multicenter cross-sectional study
Source: BMC Cancer. 2021 Sep 16;21:1034. doi: 10.1186/s12885-021-08764-x (PMC8444572; doi:10.1186/s12885-021-08764-x)
Supplement: Supplementary file 4 — Additional file 4. [file 12885_2021_8764_MOESM4_ESM.doc]

Supplementary table 2 The results of unconditional univariable logistic analysis

| **Factors** | **Esophagitis**  **OR (95% CI)** | ***P* value** | **LGIN**  **OR (95% CI)** | ***P* value** | **HGIN/ESCC**  **OR (95% CI)** | ***P* value** |
| --- | --- | --- | --- | --- | --- | --- |
| **Gender** |  |  |  |  |  |  |
| Male | 1.00 (reference) | - | 1.00 (reference) | - | 1.00 (reference) | - |
| Female | 0.80 (0.76-0.85) | <0.001 | 0.74 (0.68-0.82) | <0.001 | 0.54 (0.45-0.66) | <0.001 |
| **Age** |  |  |  |  |  |  |
| 40-49 | 1.00 (reference) | - | 1.00 (reference) | - | 1.00 (reference) | - |
| 50-59 | 1.80 (1.65-1.96) | <0.001 | 3.17 (2.65-3.81) | <0.001 | 4.96 (3.12-8.40) | <0.001 |
| 60-69 | 2.84 (2.60-3.10) | <0.001 | 7.18 (6.04-8.60) | <0.001 | 15.73 (10.06-26.29) | <0.001 |
| **Marital status** |  |  |  |  |  |  |
| Married | 1.00 (reference) | - | 1.00 (reference) | - | 1.00 (reference) | - |
| Live alone | 1.18 (1.02-1.36) | 0.021 | 1.59 (1.30-1.92) | <0.001 | 2.14 (1.49-2.97) | <0.001 |
| **Education** |  |  |  |  |  |  |
| No formal | 1.00 (reference) | - | 1.00 (reference) | - | 1.00 (reference) | - |
| Formal | 0.93 (0.86-1.01) | 0.097 | 0.68 (0.61-0.77) | <0.001 | 0.73 (0.58-0.94) | 0.013 |
| **Occupation** |  |  |  |  |  |  |
| Non-farmer | 1.00 (reference) | - | 1.00 (reference) | - | 1.00 (reference) | - |
| Farmer | 1.24 (1.15-1.33) | <0.001 | 1.29 (1.15-1.45) | <0.001 | 1.35 (1.07-1.72) | 0.014 |
| **Pesticide exposure** |  |  |  |  |  |  |
| No | 1.00 (reference) | - | 1.00 (reference) | - | 1.00 (reference) | - |
| Yes | 1.04 (0.95-1.13) | 0.435 | 1.23 (1.08-1.40) | <0.001 | 1.46 (1.13-1.87) | 0.003 |
| **Annual income per family (RMB)** |  |  |  |  |  |  |
| <10,000 | 1.00 (reference) | - | 1.00 (reference) | - | 1.00 (reference) | - |
| 10,000- | 0.86 (0.78-0.95) | 0.005 | 1.00 (0.86-1.18) | 0.973 | 0.65 (0.49-0.89) | 0.005 |
| 30,000- | 1.02 (0.93-1.13) | 0.564 | 1.03 (0.88-1.20) | 0.725 | 0.80 (0.61-1.07) | 0.124 |
| 50,000- | 0.87 (0.78-0.98) | 0.018 | 0.82 (0.69-0.99) | 0.033 | 0.60 (0.43-0.85) | 0.004 |
| ≥70,000 | 0.90 (0.79-1.02) | 0.106 | 0.58 (0.46-0.73) | <0.001 | 0.42 (0.26-0.65) | <0.001 |
| **BMI** |  |  |  |  |  |  |
| 18.5 to 23.9 | 1.00 (reference) | - | 1.00 (reference) | - | 1.00 (reference) | - |
| < 18.5 | 1.02 (0.82-1.26) | 0.878 | 0.95 (0.67-1.32) | 0.791 | 1.69 (0.97-2.73) | 0.044 |
| 24.0 to 27.9 | 0.97 (0.91-1.04) | 0.379 | 0.93 (0.84-1.03) | 0.166 | 0.80 (0.65-0.98) | 0.029 |
| ≥ 28.0 | 0.78 (0.70-0.86) | <0.001 | 0.76 (0.64-0.89) | 0.001 | 0.55 (0.37-0.78) | 0.001 |
| **Smoking** |  |  |  |  |  |  |
| Not smoke | 1.00 (reference) | - | 1.00 (reference) | - | 1.00 (reference) | - |
| Former/current smoke | 1.25 (1.16-1.33) | <0.001 | 1.28 (1.15-1.43) | <0.001 | 1.97 (1.62-2.41) | <0.001 |
| **Alcohol** |  |  |  |  |  |  |
| Not drink | 1.00 (reference) | - | 1.00 (reference) | - | 1.00 (reference) | - |
| Former/current drink | 1.22 (1.10-1.34) | <0.001 | 1.54 (1.34-1.76) | <0.001 | 2.15 (1.68-2.75) | <0.001 |
| **Tea drinking frequency** |  |  |  |  |  |  |
| Not drink | 1.00 (reference) | - | 1.00 (reference) | - | 1.00 (reference) | - |
| Former/current drink | 0.78 (0.73-0.83) | <0.001 | 1.43 (1.30-1.57) | <0.001 | 1.35 (1.11-1.65) | 0.003 |
|  |  |  |  |  |  |  |
| Supplementary table 2 The results of unconditional univariable logistic analysis (continued) | | | | | | |
| **Factors** | **Esophagitis**  **OR (95% CI)** | ***P* value** | **LGIN**  **OR (95% CI)** | ***P* value** | **HGIN/ESCC**  **OR (95% CI)** | ***P* value** |
| **Tea temperature ^a^** |  |  |  |  |  |  |
| Warm | 1.00 (reference) | - | 1.00 (reference) | - | 1.00 (reference) | - |
| Hot/burning hot tea | 1.10 (0.95-1.27) | 0.202 | 1.11 (0.92-1.33) | 0.277 | 1.34 (0.91-1.99) | 0.144 |
| **Source of drinking water** |  |  |  |  |  |  |
| Tap/pure water | 1.00 (reference) | - | 1.00 (reference) | - | 1.00 (reference) | - |
| Well water and surface water | 1.59 (1.50-1.69) | <0.001 | 1.25 (1.13-1.37) | <0.001 | 1.64 (1.35-1.98) | <0.001 |
| **Drink improved water** |  |  |  |  |  |  |
| No | 1.00 (reference) | - | 1.00 (reference) | - | 1.00 (reference) | - |
| Yes | 0.72 (0.64-0.82) | <0.001 | 0.56 (0.44-0.69) | <0.001 | 0.74 (0.49-1.08) | 0.137 |
| **Livestock meat** |  |  |  |  |  |  |
| No | 1.00 (reference) | - | 1.00 (reference) | - | 1.00 (reference) | - |
| Yes | 0.83 (0.76-0.89) | <0.001 | 1.00 (0.88-1.14) | 0.988 | 0.90 (0.71-1.18) | 0.440 |
| **Poultry meat** |  |  |  |  |  |  |
| No | 1.00 (reference) | - | 1.00 (reference) | - | 1.00 (reference) | - |
| Yes | 0.59 (0.56-0.64) | <0.001 | 0.66 (0.59-0.73) | <0.001 | 0.77 (0.63-0.95) | 0.014 |
| **Seafood** |  |  |  |  |  |  |
| No | 1.00 (reference) | - | 1.00 (reference) | - | 1.00 (reference) | - |
| Yes | 0.86 (0.80-0.94) | <0.001 | 1.08 (0.95-1.21) | 0.220 | 0.99 (0.77-1.27) | 0.967 |
| **Fruits** |  |  |  |  |  |  |
| No | 1.00 (reference) | - | 1.00 (reference) | - | 1.00 (reference) | - |
| Yes | 0.78 (0.73-0.83) | <0.001 | 0.79 (0.71-0.87) | <0.001 | 0.70 (0.58-0.85) | <0.001 |
| **Bean products** |  |  |  |  |  |  |
| No | 1.00 (reference) | - | 1.00 (reference) | - | 1.00 (reference) | - |
| Yes | 1.06 (1.00-1.12) | 0.067 | 1.04 (0.95-1.14) | 0.435 | 1.06 (0.88-1.28) | 0.534 |
| **Spring onion/ginger/garlic** |  |  |  |  |  |  |
| No | 1.00 (reference) | - | 1.00 (reference) | - | 1.00 (reference) | - |
| Yes | 0.68 (0.64-0.72) | <0.001 | 0.88 (0.80-0.97) | 0.009 | 0.78 (0.64-0.95) | 0.014 |
| **Nut** |  |  |  |  |  |  |
| No | 1.00 (reference) | - | 1.00 (reference) | - | 1.00 (reference) | - |
| Yes | 0.74 (0.68-0.80) | <0.001 | 0.83 (0.73-0.94) | 0.003 | 0.93 (0.72-1.18) | 0.537 |
| **Milk** |  |  |  |  |  |  |
| No | 1.00 (reference) | - | 1.00 (reference) | - | 1.00 (reference) | - |
| Yes | 0.99 (0.91-1.07) | 0.828 | 0.81 (0.71-0.93) | 0.003 | 0.99 (0.76-1.27) | 0.937 |
| **Soybean milk** |  |  |  |  |  |  |
| No | 1.00 (reference) | - | 1.00 (reference) | - | 1.00 (reference) | - |
| Yes | 0.81 (0.69-0.94) | 0.006 | 0.71 (0.55-0.91) | 0.009 | 0.72 (0.41-1.16) | 0.209 |
|  |  |  |  |  |  |  |
|  |  |  |  |  |  |  |
|  |  |  |  |  |  |  |
| Supplementary table 2 The results of unconditional univariable logistic analysis (continued) | | | | | | |
| **Factors** | **Esophagitis**  **OR (95% CI)** | ***P* value** | **LGIN**  **OR (95% CI)** | ***P* value** | **HGIN/ESCC**  **OR (95% CI)** | ***P* value** |
| **Vitamins** |  |  |  |  |  |  |
| No | 1.00 (reference) | - | 1.00 (reference) | - | 1.00 (reference) | - |
| Yes | 0.64 (0.47-0.85) | 0.003 | 0.43 (0.24-0.72) | 0.003 | 0.72 (0.26-1.56) | 0.464 |
| **Leftovers** |  |  |  |  |  |  |
| No | 1.00 (reference) | - | 1.00 (reference) | - | 1.00 (reference) | - |
| Yes | 1.46 (1.38-1.55) | <0.001 | 1.07 (0.98-1.18) | 0.150 | 1.16 (0.96-1.40) | 0.119 |
| **Eat out** |  |  |  |  |  |  |
| No | 1.00 (reference) | - | 1.00 (reference) | - | 1.00 (reference) | - |
| Yes | 0.97 (0.88-1.06) | 0.505 | 0.75 (0.64-0.87) | <0.001 | 0.94 (0.69-1.25) | 0.670 |
| **Diet taste** |  |  |  |  |  |  |
| Light diet | 1.00 (reference) | - | 1.00 (reference) | - | 1.00 (reference) | - |
| Salty diet | 1.59 (1.47-1.74) | <0.001 | 1.60 (1.41-1.83) | <0.001 | 1.40 (1.09-1.81) | 0.009 |
| **Refrigerator** |  |  |  |  |  |  |
| No refrigerator | 1.00 (reference) | - | 1.00 (reference) | - | 1.00 (reference) | - |
| 1-10years | 0.66 (0.60-0.74) | <0.001 | 0.74 (0.63-0.87) | <0.001 | 0.57 (0.43-0.78) | <0.001 |
| 11-20years | 0.61 (0.54-0.69) | <0.001 | 0.64 (0.52-0.78) | <0.001 | 0.52 (0.36-0.76) | 0.001 |
| >20years | 0.48 (0.32-0.69) | <0.001 | 0.60 (0.33-1.01) | 0.070 | 0.44 (0.11-1.21) | 0.170 |
| **Cooking** |  |  |  |  |  |  |
| No | 1.00 (reference) | - | 1.00 (reference) | - | 1.00 (reference) | - |
| Yes | 0.82 (0.77-0.88) | <0.001 | 0.79 (0.72-0.88) | <0.001 | 0.75 (0.62-0.92) | 0.005 |
| **Physical exercise** |  |  |  |  |  |  |
| No | 1.00 (reference) | - | 1.00 (reference) | - | 1.00 (reference) | - |
| Yes | 0.67 (0.61-0.74) | <0.001 | 0.85 (0.74-0.98) | 0.023 | 0.94 (0.71-1.23) | 0.675 |
| **Housework** |  |  |  |  |  |  |
| No | 1.00 (reference) | - | 1.00 (reference) | - | 1.00 (reference) | - |
| <8h/week | 0.59 (0.54-0.65) | <0.001 | 0.86 (0.73-1.01) | 0.057 | 0.97 (0.71-1.34) | 0.859 |
| 8-14h/week | 0.54 (0.49-0.59) | <0.001 | 0.79 (0.67-0.93) | 0.004 | 0.78 (0.57-1.08) | 0.123 |
| 15-21h/week | 0.56 (0.50-0.62) | <0.001 | 0.78 (0.65-0.93) | 0.006 | 0.80 (0.56-1.15) | 0.217 |
| ≥22h/week | 0.61 (0.55-0.68) | <0.001 | 0.98 (0.83-1.17) | 0.843 | 0.65 (0.44-0.96) | 0.029 |
| **Number of teeth lost** |  |  |  |  |  |  |
| Never | 1.00 (reference) | - | 1.00 (reference) | - | 1.00 (reference) | - |
| 1-3 | 1.24 (1.16-1.33) | <0.001 | 1.38 (1.24-1.54) | <0.001 | 1.07 (0.84-1.36) | 0.570 |
| 4-6 | 1.35 (1.22-1.48) | <0.001 | 1.53 (1.31-1.77) | <0.001 | 1.68 (1.26-2.22) | <0.001 |
| 7-11 | 1.61 (1.41-1.82) | <0.001 | 1.88 (1.55-2.26) | <0.001 | 1.54 (1.01-2.26) | 0.035 |
| 12-31 | 2.05 (1.76-2.37) | <0.001 | 2.18 (1.73-2.72) | <0.001 | 2.62 (1.70-3.87) | <0.001 |
| Complete denture | 2.66 (2.30-3.07) | <0.001 | 2.55 (2.02-3.19) | <0.001 | 2.72 (1.72-4.10) | <0.001 |
| **Snore** |  |  |  |  |  |  |
| No | 1.00 (reference) | - | 1.00 (reference) | - | 1.00 (reference) | - |
| Yes | 1.11 (1.04-1.18) | 0.001 | 1.07 (0.97-1.17) | 0.172 | 1.11 (0.92-1.34) | 0.273 |
|  |  |  |  |  |  |  |
|  |  |  |  |  |  |  |
| Supplementary table 2 The results of unconditional univariable logistic analysis (continued) | | | | | | |
| Factors | Esophagitis  OR (95% CI) | *P* value | LGIN  OR (95% CI) | *P* value | HGIN/ESCC  OR (95% CI) | *P* value |
| **Nap** |  |  |  |  |  |  |
| No | 1.00 (reference) | - | 1.00 (reference) | - | 1.00 (reference) | - |
| Yes | 0.98 (0.92-1.05) | 0.640 | 0.86 (0.78-0.95) | 0.003 | 0.83 (0.68-1.01) | 0.059 |
| **Loose teeth** |  |  |  |  |  |  |
| No | 1.00 (reference) | - | 1.00 (reference) | - | 1.00 (reference) | - |
| Yes | 1.23 (1.06-1.42) | 0.005 | 1.49 (1.21-1.82) | <0.001 | 1.89 (1.27-2.70) | 0.001 |
| **History of chronic hepatitis and cirrhosis** |  |  |  |  |  |  |
| No | 1.00 (reference) | - | 1.00 (reference) | - | 1.00 (reference) | - |
| Yes | 3.05 (2.60-3.57) | <0.001 | 1.36 (0.96-1.86) | 0.067 | 1.96 (1.07-3.28) | 0.018 |
| **Family history of cancer** |  |  |  |  |  |  |
| No | 1.00 (reference) | - | 1.00 (reference) | - | 1.00 (reference) | - |
| Yes | 1.19 (1.11-1.27) | <0.001 | 1.24 (1.12-1.37) | <0.001 | 1.50 (1.23-1.82) | <0.001 |
| **Take an acid suppressant** |  |  |  |  |  |  |
| No | 1.00 (reference) | - | 1.00 (reference) | - | 1.00 (reference) | - |
| Yes | 0.68 (0.59-0.79) | <0.001 | 0.49 (0.37-0.63) | <0.001 | 0.65 (0.39-1.02) | 0.077 |
| **Take antibiotics** |  |  |  |  |  |  |
| Not take | 1.00 (reference) | - | 1.00 (reference) | - | 1.00 (reference) | - |
| Not every week | 0.54 (0.40-0.73) | <0.001 | 0.76 (0.12-0.72) | 0.180 | 0.27 (0.07-1.08) | 0.063 |
| Not every day | 0.70 (0.461.08) | 0.107 | 0.80 (0.43-1.51) | 0.492 | 0.68 (0.17-2.74) | 0.588 |
| Every day | 0.39 (0.24-0.63) | <0.001 | 0.30 (0.12-0.72) | 0.007 | 0.25 (0.04-1.80) | 0.169 |

**Legend:** ^a^ = Only part of the data with tea drinking temperature was analyzed, not all. BMI = body mass index (kg/m^2^). LGIN = low-grade intraepithelial neoplasia; HGIN = high-grade intraepithelial neoplasia; ESCC = esophageal squamous cell carcinoma. Source: WHO tumor histological classification 2000.
