# Supplementary material for: Risk factors for esophageal squamous cell carcinoma and its histological precursor lesions in China: a multicenter cross-sectional study
Source: BMC Cancer. 2021 Sep 16;21:1034. doi: 10.1186/s12885-021-08764-x (PMC8444572; doi:10.1186/s12885-021-08764-x)
Supplement: Supplementary file 5 — Additional file 5. [file 12885_2021_8764_MOESM5_ESM.doc]

Supplementary table 3 The results of unconditional multivariable logistic analysis for Esophagitis

| **Factors** | **Age-adjusted**  **OR (95% CI)** | ***P* value** | **Gender-adjusted**  **OR (95% CI)** | ***P* value** | **Adjusted OR ^a^**  **(95% CI)** | ***P* value** |
| --- | --- | --- | --- | --- | --- | --- |
| **Marital status** |  |  |  |  |  |  |
| Married | 1.00 (reference) | - | 1.00 (reference) | - | 1.00 (reference) | - |
| Live alone | 0.96 (0.83-1.11) | 0.580 | 1.19 (1.03-1.37) | 0.016 | 1.02 (0.88-1.17) | 0.802 |
| **Occupation** |  |  |  |  |  |  |
| Non-farmer | 1.00 (reference) | - | 1.00 (reference) | - | 1.00 (reference) | - |
| Farmer | 1.20 (1.11-1.29) | <0.001 | 1.24 (1.15-1.34) | <0.001 | 1.21 (1.12-1.30) | <0.001 |
| **Smoking** |  |  |  |  |  |  |
| Not smoke | 1.00 (reference) | - | 1.00 (reference) | - | 1.00 (reference) | - |
| Former/current smoke | 1.19 (1.11-1.28) | <0.001 | 1.18 (1.10-1.28) | <0.001 | 1.09 (1.00-1.19) | 0.048 |
| **Alcohol** |  |  |  |  |  |  |
| Not drink | 1.00 (reference) | - | 1.00 (reference) | - | 1.00 (reference) | - |
| Former/current drink | 1.16 (1.05-1.28) | 0.003 | 0.82 (0.77-0.87) | <0.001 | 1.05 (0.95-1.17) | 0.331 |
| **Tea drinking frequency** |  |  |  |  |  |  |
| Not drink | 1.00 (reference) | - | 1.00 (reference) | - | 1.00 (reference) | - |
| Former/current drink | 0.77 (0.72-0.83) | <0.001 | 0.79 (0.75-0.84) | <0.001 | 0.76 (0.70-0.81) | <0.001 |
| **Tea temperature ^b^** |  |  |  |  |  |  |
| Warm | 1.00 (reference) | - | 1.00 (reference) | - | 1.00 (reference) | - |
| Hot/burning hot tea | 1.09 (0.95-1.26) | 0.231 | 0.61 (0.54-0.69) | <0.001 | 1.12 (0.97-1.29) | 0.137 |
| **Source of drinking water** |  |  |  |  |  |  |
| Tap/pure water | 1.00 (reference) | - | 1.00 (reference) | - | 1.00 (reference) | - |
| Well water and surface water | 1.53 (1.44-1.62) | <0.001 | 1.59 (1.50-1.69) | <0.001 | 1.61 (1.51-1.71) | <0.001 |
| **Drink improved water** |  |  |  |  |  |  |
| No | 1.00 (reference) | - | 1.00 (reference) | - | 1.00 (reference) | - |
| Yes | 0.73 (0.64-0.83) | <0.001 | 0.72 (0.64-0.82) | <0.001 | 0.73 (0.64-0.83) | <0.001 |
| **Livestock meat** |  |  |  |  |  |  |
| No | 1.00 (reference) | - | 1.00 (reference) | - | 1.00 (reference) | - |
| Yes | 0.89 (0.82-0.96) | 0.004 | 0.81 (0.75-0.88) | <0.001 | 0.88 (0.81-0.95) | 0.001 |
| **Poultry meat** |  |  |  |  |  |  |
| No | 1.00 (reference) | - | 1.00 (reference) | - | 1.00 (reference) | - |
| Yes | 0.64 (0.60-0.69) | <0.001 | 0.59 (0.55-0.63) | <0.001 | 0.62 (0.58-0.67) | <0.001 |
| **Seafood** |  |  |  |  |  |  |
| No | 1.00 (reference) | - | 1.00 (reference) | - | 1.00 (reference) | - |
| Yes | 0.89 (0.82-0.96) | 0.004 | 0.86 (0.79-0.94) | <0.001 | 0.89 (0.82-0.97) | 0.007 |
| **Fruits** |  |  |  |  |  |  |
| No | 1.00 (reference) | - | 1.00 (reference) | - | 1.00 (reference) | - |
| Yes | 0.83 (0.78-0.88) | <0.001 | 0.79 (0.74-0.84) | <0.001 | 0.83 (0.78-0.88) | <0.001 |
|  |  |  |  |  |  |  |
|  |  |  |  |  |  |  |
|  |  |  |  |  |  |  |
| Supplementary table 3 The results of unconditional multivariable logistic analysis for Esophagitis (continued) | | | | | | |
| **Factors** | **Age-adjusted**  **OR (95% CI)** | ***P* value** | **Gender-adjusted**  **OR (95% CI)** | ***P* value** | **Adjusted OR ^a^**  **(95% CI)** | ***P* value** |
| **Bean products** |  |  |  |  |  |  |
| No | 1.00 (reference) | - | 1.00 (reference) | - | 1.00 (reference) | - |
| Yes | 1.09 (1.03-1.16) | 0.005 | 1.06 (0.99-1.12) | 0.076 | 1.09 (1.03-1.16) | 0.005 |
| **Spring onion/ginger/garlic** |  |  |  |  |  |  |
| No | 1.00 (reference) | - | 1.00 (reference) | - | 1.00 (reference) | - |
| Yes | 0.70 (0.66-0.74) | <0.001 | 0.68 (0.64-0.72) | <0.001 | 0.69 (0.65-0.74) | <0.001 |
| **Nuts** |  |  |  |  |  |  |
| No | 1.00 (reference) | - | 1.00 (reference) | - | 1.00 (reference) | - |
| Yes | 0.76 (0.70-0.83) | <0.001 | 0.73 (0.67-0.80) | <0.001 | 0.77 (0.71-0.84) | <0.001 |
| **Soybean milk** |  |  |  |  |  |  |
| No | 1.00 (reference) | - | 1.00 (reference) | - | 1.00 (reference) | - |
| Yes | 0.88 (0.75-1.02) | 0.093 | 0.81 (0.69-0.94) | 0.006 | 0.87 (0.74-1.01) | 0.076 |
| **Vitamins** |  |  |  |  |  |  |
| No | 1.00 (reference) | - | 1.00 (reference) | - | 1.00 (reference) | - |
| Yes | 0.69 (0.51-0.91) | 0.011 | 0.64 (0.48-0.85) | 0.003 | 0.68 (0.50-0.90) | 0.010 |
| **Leftovers** |  |  |  |  |  |  |
| No | 1.00 (reference) | - | 1.00 (reference) | - | 1.00 (reference) | - |
| Yes | 1.44 (1.35-1.53) | <0.001 | 1.47 (1.39-1.56) | <0.001 | 1.49 (1.40-1.58) | <0.001 |
| **Diet taste** |  |  |  |  |  |  |
| Light diet | 1.00 (reference) | - | 1.00 (reference) | - | 1.00 (reference) | - |
| Salty diet | 1.59 (1.46-1.72) | <0.001 | 1.59 (1.47-1.73) | <0.001 | 1.57 (1.45-1.71) | <0.001 |
| **Refrigerator** |  |  |  |  |  |  |
| No refrigerator | 1.00 (reference) | - | 1.00 (reference) | - | 1.00 (reference) | - |
| 1-10years | 0.75 (0.68-0.83) | <0.001 | 0.67 (0.60-0.74) | <0.001 | 0.73 (0.66-0.81) | <0.001 |
| 11-20years | 0.70 (0.62-0.79) | <0.001 | 0.61 (0.54-0.69) | <0.001 | 0.67 (0.59-0.77) | <0.001 |
| >20years | 0.53 (0.36-0.76) | 0.001 | 0.47 (0.32-0.67) | <0.001 | 0.50 (0.33-0.71) | <0.001 |
| **Cooking** |  |  |  |  |  |  |
| No | 1.00 (reference) | - | 1.00 (reference) | - | 1.00 (reference) | - |
| Yes | 0.84 (0.78-0.89) | <0.001 | 0.94 (0.86-1.02) | 0.156 | 0.92 (0.84-1.00) | 0.063 |
| **Take acid suppressants** |  |  |  |  |  |  |
| No | 1.00 (reference) | - | 1.00 (reference) | - | 1.00 (reference) | - |
| Yes | 0.70 (0.60-0.81) | <0.001 | 0.67 (0.58-0.77) | <0.001 | 0.70 (0.60-0.80) | <0.001 |
| **Housework** |  |  |  |  |  |  |
| No | 1.00 (reference) | - | 1.00 (reference) | - | 1.00 (reference) | - |
| <8h/week | 0.60 (0.54-0.66) | <0.001 | 0.61 (0.55-0.67) | <0.001 | 0.61 (0.56-0.68) | <0.001 |
| 8-14h/week | 0.55 (0.50-0.60) | <0.001 | 0.57 (0.51-0.62) | <0.001 | 0.57 (0.52-0.63) | <0.001 |
| 15-21h/week | 0.55 (0.49-0.61) | <0.001 | 0.59 (0.53-0.66) | <0.001 | 0.58 (0.52-0.65) | <0.001 |
| ≥22h/week | 0.60 (0.54-0.67) | <0.001 | 0.65 (0.58-0.73) | <0.001 | 0.63 (0.56-0.71) | <0.001 |
|  |  |  |  |  |  |  |
| Supplementary table 3 The results of unconditional multivariable logistic analysis for Esophagitis (continued) | | | | | | |
| **Factors** | **Age-adjusted**  **OR (95% CI)** | ***P* value** | **Gender-adjusted**  **OR (95% CI)** | ***P* value** | **Adjusted OR ^a^**  **(95% CI)** | ***P* value** |
| **Physical exercise** |  |  |  |  |  |  |
| No | 1.00 (reference) | - | 1.00 (reference) | - | 1.00 (reference) | - |
| Yes | 0.68 (0.62-0.75) | <0.001 | 0.68 (0.61-0.74) | <0.001 | 0.69 (0.62-0.76) | <0.001 |
| **Snore** |  |  |  |  |  |  |
| No | 1.00 (reference) | - | 1.00 (reference) | - | 1.00 (reference) | - |
| Yes | 1.10 (1.03-1.16) | 0.003 | 1.07 (1.01-1.14) | 0.025 | 1.09 (1.02-1.15) | 0.009 |
| **Loose teeth** |  |  |  |  |  |  |
| No | 1.00 (reference) | - | 1.00 (reference) | - | 1.00 (reference) | - |
| Yes | 1.11 (0.96-1.28) | 0.158 | 1.21 (1.04-1.39) | 0.010 | 1.10 (0.95-1.27) | 0.202 |
| **Number of teeth lost** |  |  |  |  |  |  |
| Never | 1.00 (reference) | - | 1.00 (reference) | - | 1.00 (reference) | - |
| 1-3 | 1.13 (1.05-1.21) | 0.001 | 1.24 (1.15-1.33) | <0.001 | 1.14 (1.06-1.23) | <0.001 |
| 4-6 | 1.12 (1.02-1.24) | 0.021 | 1.35 (1.23-1.49) | <0.001 | 1.15 (1.04-1.27) | 0.005 |
| 7-11 | 1.23 (1.08-1.40) | 0.002 | 1.62 (1.42-1.83) | <0.001 | 1.27 (1.12-1.45) | <0.001 |
| 12-31 | 1.47 (1.26-1.71) | <0.001 | 2.05 (1.76-2.37) | <0.001 | 1.54 (1.32-1.79) | <0.001 |
| Complete denture | 1.85 (1.59-2.14) | <0.001 | 2.69 (2.32-3.10) | <0.001 | 1.94 (1.66-2.25) | <0.001 |
| **History of chronic hepatitis** |  |  |  |  |  |  |
| No | 1.00 (reference) | - | 1.00 (reference) | - | 1.00 (reference) | - |
| Yes | 3.01 (2.56-3.53) | <0.001 | 3.05 (2.60-3.56) | <0.001 | 2.96 (2.52-3.47) | <0.001 |
| **Family history of cancer** |  |  |  |  |  |  |
| No | 1.00 (reference) | - | 1.00 (reference) | - | 1.00 (reference) | - |
| Yes | 1.22 (1.14-1.30) | <0.001 | 1.18 (1.11-1.26) | <0.001 | 1.22 (1.14-1.30) | <0.001 |
| **Take antibiotics** |  |  |  |  |  |  |
| Not take | 1.00 (reference) | - | 1.00 (reference) | - | 1.00 (reference) | - |
| Not every week | 0.58 (0.43-0.79) | 0.001 | 0.54 (0.75-0.85) | <0.001 | 0.61 (0.45-0.83) | 0.001 |
| Not every day | 0.75 (0.49-1.16) | 0.193 | 0.70 (0.45-1.07) | 0.096 | 0.75 (0.49-1.16) | 0.197 |
| Every day | 0.42 (0.26-0.68) | <0.001 | 0.38 (0.23-0.61) | <0.001 | 0.40 (0.24-0.65) | <0.001 |

**Legend:** ^a^ Adjusted for age, gender, education, BMI and income. ^b^ = Only part of the data with tea drinking temperature was analyzed, not all.

Supplementary table 4 The results of unconditional multivariable logistic analysis for low-grade intraepithelial neoplasia

| **Factors** | **Age-adjusted**  **OR (95% CI)** | ***P* value** | **Gender-adjusted**  **OR (95% CI)** | ***P* value** | **Adjusted OR ^a^**  **(95% CI)** | ***P* value** |
| --- | --- | --- | --- | --- | --- | --- |
| **Marital status** |  |  |  |  |  |  |
| Married | 1.00 (reference) | - | 1.00 (reference) | - | 1.00 (reference) | - |
| Live alone | 1.13 (0.92-1.37) | 0.223 | 1.60 (1.31-1.93) | <0.001 | 1.22 (1.00-1.48) | 0.049 |
| **Occupation** |  |  |  |  |  |  |
| Non-farmer | 1.00 (reference) | - | 1.00 (reference) | - | 1.00 (reference) | - |
| Farmer | 1.22 (1.09-1.38) | 0.001 | 1.29 (1.15-1.45) | <0.001 | 1.20 (1.07-1.35) | 0.002 |
| **Pesticide exposure** |  |  |  |  |  |  |
| No | 1.00 (reference) | - | 1.00 (reference) | - | 1.00 (reference) | - |
| Yes | 1.20 (1.05-1.36) | 0.008 | 1.25 (1.09-1.42) | 0.001 | 1.20 (1.05-1.37) | 0.007 |
| **Smoking** |  |  |  |  |  |  |
| Not smoke | 1.00 (reference) | - | 1.00 (reference) | - | 1.00 (reference) | - |
| Former/current smoke | 1.19 (1.07-1.32) | 0.002 | 1.05 (0.94-1.22) | 0.324 | 1.04 (0.91-1.19) | 0.574 |
| **Alcohol** |  |  |  |  |  |  |
| Not drink | 1.00 (reference) | - | 1.00 (reference) | - | 1.00 (reference) | - |
| Former/current drink | 1.42 (1.24-1.63) | <0.001 | 1.35 (1.17-1.56) | <0.001 | 1.30 (1.12-1.51) | <0.001 |
| **Tea drinking frequency** |  |  |  |  |  |  |
| Not drink | 1.00 (reference) | - | 1.00 (reference) | - | 1.00 (reference) | - |
| Former/current drink | 1.42 (1.29-1.57) | <0.001 | 1.40 (1.27-1.55) | <0.001 | 1.34 (1.26-1.54) | <0.001 |
| **Tea temperature ^b^** |  |  |  |  |  |  |
| Warm | 1.00 (reference) | - | 1.00 (reference) | - | 1.00 (reference) | - |
| Hot/burning hot tea | 1.09 (0.91-1.31) | 0.345 | 1.10 (0.92-1.32) | 0.295 | 1.10 (0.92-1.33) | 0.300 |
| **Source of drinking water** |  |  |  |  |  |  |
| Tap/pure water | 1.00 (reference) | - | 1.00 (reference) | - | 1.00 (reference) | - |
| Well water and surface water | 1.16 (1.06-1.28) | 0.002 | 1.25 (1.13-1.37) | <0.001 | 1.19 (1.08-1.32) | <0.001 |
| **Drink improved water** |  |  |  |  |  |  |
| No | 1.00 (reference) | - | 1.00 (reference) | - | 1.00 (reference) | - |
| Yes | 0.57 (0.45-0.70) | <0.001 | 0.56 (0.44-0.69) | <0.001 | 0.58 (0.46-0.72) | <0.001 |
| **Poultry meat** |  |  |  |  |  |  |
| No | 1.00 (reference) | - | 1.00 (reference) | - | 1.00 (reference) | - |
| Yes | 0.75 (0.67-0.83) | <0.001 | 0.64 (0.58-0.71) | <0.001 | 0.73 (0.66-0.81) | <0.001 |
| **Fruits** |  |  |  |  |  |  |
| No | 1.00 (reference) | - | 1.00 (reference) | - | 1.00 (reference) | - |
| Yes | 0.88 (0.80-0.97) | 0.008 | 0.80 (0.73-0.89) | <0.001 | 0.89 (0.80-0.98) | 0.015 |
| **Nut** |  |  |  |  |  |  |
| No | 1.00 (reference) | - | 1.00 (reference) | - | 1.00 (reference) | - |
| Yes | 0.87 (0.77-0.99) | 0.035 | 0.82 (0.72-0.93) | 0.002 | 0.89 (0.78-1.01) | 0.067 |
|  |  |  |  |  |  |  |
| Supplementary table 4 The results of unconditional multivariable logistic analysis for low-grade intraepithelial neoplasia (continued) | | | | | | |
| **Factors** | **Age-adjusted**  **OR (95% CI)** | ***P* value** | **Gender-adjusted**  **OR (95% CI)** | ***P* value** | **Adjusted OR ^a^**  **(95% CI)** | ***P* value** |
| **Spring onion/ginger/garlic** |  |  |  |  |  |  |
| No | 1.00 (reference) | - | 1.00 (reference) | - | 1.00 (reference) | - |
| Yes | 0.91 (0.83-1.01) | 0.076 | 0.87 (0.79-0.96) | 0.006 | 0.93 (0.85-1.03) | 0.188 |
| **Milk** |  |  |  |  |  |  |
| No | 1.00 (reference) | - | 1.00 (reference) | - | 1.00 (reference) | - |
| Yes | 0.81 (0.70-0.92) | 0.002 | 0.80 (0.70-0.92) | 0.001 | 0.82 (0.71-0.94) | 0.004 |
| **Soybean milk** |  |  |  |  |  |  |
| No | 1.00 (reference) | - | 1.00 (reference) | - | 1.00 (reference) | - |
| Yes | 0.81 (0.62-1.04) | 0.113 | 0.71 (0.55-0.92) | 0.009 | 0.84 (0.65-1.08) | 0.198 |
| **Vitamins** |  |  |  |  |  |  |
| No | 1.00 (reference) | - | 1.00 (reference) | - | 1.00 (reference) | - |
| Yes | 0.48 (0.26-0.80) | 0.010 | 0.44 (0.24-0.72) | 0.003 | 0.49 (0.27-0.82) | 0.012 |
| **Leftovers** |  |  |  |  |  |  |
| No | 1.00 (reference) | - | 1.00 (reference) | - | 1.00 (reference) | - |
| Yes | 1.04 (0.94-1.14) | 0.438 | 1.08 (0.98-1.18) | 0.112 | 1.08 (0.98-1.19) | 0.108 |
| **Eat out** |  |  |  |  |  |  |
| No | 1.00 (reference) | - | 1.00 (reference) | - | 1.00 (reference) | - |
| Yes | 0.96 (0.81-1.12) | 0.581 | 0.70 (0.60-0.82) | <0.001 | 0.92 (0.78-1.07) | 0.286 |
| **Diet taste** |  |  |  |  |  |  |
| Light diet | 1.00 (reference) | - | 1.00 (reference) | - | 1.00 (reference) | - |
| Salty diet | 1.59 (1.40-1.81) | <0.001 | 1.60 (1.41-1.82) | <0.001 | 1.57 (1.38-1.80) | <0.001 |
| **Refrigerator** |  |  |  |  |  |  |
| No refrigerator | 1.00 (reference) | - | 1.00 (reference) | - | 1.00 (reference) | - |
| 1-10years | 0.92 (0.78-1.08) | 0.301 | 0.74 (0.63-0.87) | <0.001 | 0.88 (0.75-1.04) | 0.135 |
| 11-20years | 0.80 (0.66-0.98) | 0.032 | 0.63 (0.52-0.77) | <0.001 | 0.78 (0.64-0.95) | 0.015 |
| >20years | 0.71 (0.39-1.20) | 0.236 | 0.58 (0.32-0.97) | 0.053 | 0.69 (0.37-1.16) | 0.189 |
| **Cooking** |  |  |  |  |  |  |
| No | 1.00 (reference) | - | 1.00 (reference) | - | 1.00 (reference) | - |
| Yes | 0.82 (0.74-0.90) | <0.001 | 0.97 (0.85-1.11) | 0.676 | 0.95 (0.83-1.08) | 0.411 |
| **Physical exercise** |  |  |  |  |  |  |
| No | 1.00 (reference) | - | 1.00 (reference) | - | 1.00 (reference) | - |
| Yes | 0.87 (0.76-1.00) | 0.061 | 0.86 (0.75-0.99) | 0.037 | 0.89 (0.77-1.03) | 0.119 |
| **Housework** |  |  |  |  |  |  |
| No | 1.00 (reference) | - | 1.00 (reference) | - | 1.00 (reference) | - |
| <8h/week | 0.88 (0.75-1.04) | 0.133 | 0.92 (0.78-1.08) | 0.288 | 0.92 (0.78-1.08) | 0.317 |
| 8-14h/week | 0.82 (0.70-0.96) | 0.015 | 0.90 (0.76-1.06) | 0.191 | 0.88 (0.75-1.05) | 0.147 |
| 15-21h/week | 0.76 (0.64-0.91) | 0.003 | 0.90 (0.75-1.08) | 0.257 | 0.84 (0.70-1.01) | 0.066 |
| ≥22h/week | 0.97 (0.81-1.15) | 0.705 | 1.15 (0.96-1.39) | 0.126 | 1.06 (0.88-1.27) | 0.542 |
|  |  |  |  |  |  |  |
| Supplementary table 4 The results of unconditional multivariable logistic analysis for low-grade intraepithelial neoplasia (continued) | | | | | | |
| **Factors** | **Age-adjusted**  **OR (95% CI)** | ***P* value** | **Gender-adjusted**  **OR (95% CI)** | ***P* value** | **Adjusted OR ^a^**  **(95% CI)** | ***P* value** |
| **Snore** |  |  |  |  |  |  |
| No | 1.00 (reference) | - | 1.00 (reference) | - | 1.00 (reference) | - |
| Yes | 1.05 (0.96-1.16) | 0.271 | 1.02 (0.93-1.12) | 0.714 | 1.04 (0.95-1.15) | 0.414 |
| **Nap** |  |  |  |  |  |  |
| No | 1.00 (reference) | - | 1.00 (reference) | - | 1.00 (reference) | - |
| Yes | 0.91 (0.82-1.00) | 0.056 | 0.85 (0.77-0.93) | 0.001 | 0.89 (0.81-0.99) | 0.025 |
| **Loose teeth** |  |  |  |  |  |  |
| No | 1.00 (reference) | - | 1.00 (reference) | - | 1.00 (reference) | - |
| Yes | 1.26 (1.02-1.54) | 0.031 | 1.45 (1.17-1.77) | <0.001 | 1.23 (1.00-1.51) | 0.049 |
| **Number of teeth lost** |  |  |  |  |  |  |
| Never | 1.00 (reference) | - | 1.00 (reference) | - | 1.00 (reference) | - |
| 1-3 | 1.15 (1.03-1.29) | 0.014 | 1.38 (1.23-1.54) | <0.001 | 1.17 (1.04-1.31) | 0.007 |
| 4-6 | 1.09 (0.94-1.27) | 0.253 | 1.53 (1.32-1.77) | <0.001 | 1.12 (0.96-1.30) | 0.137 |
| 7-11 | 1.14 (0.94-1.38) | 0.184 | 1.90 (1.57-2.29) | <0.001 | 1.19 (0.98-1.44) | 0.077 |
| 12-31 | 1.19 (0.93-1.49) | 0.150 | 2.18 (1.73-2.72) | <0.001 | 1.26 (0.99-1.58) | 0.054 |
| Complete denture | 1.32 (1.04-1.67) | 0.020 | 2.58 (2.04-3.23) | <0.001 | 1.42 (1.11-1.78) | 0.004 |
| **History of chronic hepatitis** |  |  |  |  |  |  |
| No | 1.00 (reference) | - | 1.00 (reference) | - | 1.00 (reference) | - |
| Yes | 1.34 (0.95-1.85) | 0.080 | 1.34 (0.95-1.83) | 0.081 | 1.36 (0.96-1.87) | 0.070 |
| **Family history of cancer** |  |  |  |  |  |  |
| No | 1.00 (reference) | - | 1.00 (reference) | - | 1.00 (reference) | - |
| Yes | 1.30 (1.17-1.44) | <0.001 | 1.24 (1.12-1.37) | <0.001 | 1.33 (1.20-1.47) | <0.001 |
| **Take acid suppressants** |  |  |  |  |  |  |
| No | 1.00 (reference) | - | 1.00 (reference) | - | 1.00 (reference) | - |
| Yes | 0.51 (0.39-0.66) | <0.001 | 0.47 (0.36-0.61) | <0.001 | 0.51 (0.39-0.66) | <0.001 |
| **Take antibiotics** |  |  |  |  |  |  |
| Not take | 1.00 (reference) | - | 1.00 (reference) | - | 1.00 (reference) | - |
| Not every week | 0.87 (0.58-1.32) | 0.517 | 0.76 (0.51-1.15) | 0.196 | 0.90 (0.59-1.36) | 0.617 |
| Not every day | 0.89 (0.47-1.69) | 0.730 | 0.79 (0.42-1.48) | 0.455 | 0.93 (0.49-1.77) | 0.826 |
| Every day | 0.34 (0.14-0.83) | 0.017 | 0.29 (0.12-0.70) | 0.006 | 0.33 (0.14-0.80) | 0.014 |

**Legend:** ^a^ Adjusted for age, gender, education, BMI and income. ^b^ = Only part of the data with tea drinking temperature was analyzed, not all. LGIN = low-grade intraepithelial neoplasia.

Supplementary table 5 The results of unconditional multivariable logistic analysis for high-grade intraepithelial neoplasia and above

| **Factors** | **Age-adjusted**  **OR (95% CI)** | ***P* value** | **Gender-adjusted**  **OR (95% CI)** | ***P* value** | **Adjusted OR ^a^**  **(95% CI)** | ***P* value** |
| --- | --- | --- | --- | --- | --- | --- |
| **Marital status** |  |  |  |  |  |  |
| Married | 1.00 (reference) | - | 1.00 (reference) | - | 1.00 (reference) | - |
| Live alone | 1.39 (0.96-1.94) | 0.064 | 2.16 (1.50-3.00) | <0.001 | 1.49 (1.03-2.10) | 0.026 |
| **Occupation** |  |  |  |  |  |  |
| Non-farmer | 1.00 (reference) | - | 1.00 (reference) | - | 1.00 (reference) | - |
| Farmer | 1.28 (1.01-1.63) | 0.043 | 1.36 (1.08-1.73) | 0.011 | 1.22 (0.97-1.57) | 0.098 |
| **Pesticide exposure** |  |  |  |  |  |  |
| No | 1.00 (reference) | - | 1.00 (reference) | - | 1.00 (reference) | - |
| Yes | 1.43 (1.11-1.83) | 0.005 | 1.51 (1.16-1.92) | 0.001 | 1.44 (1.11-1.84) | 0.005 |
| **Smoking** |  |  |  |  |  |  |
| Not smoke | 1.00 (reference) | - | 1.00 (reference) | - | 1.00 (reference) | - |
| Former/current smoke | 1.79 (1.47-1.18) | <0.001 | 1.52 (1.18-1.96) | 0.001 | 1.46 (1.14-1.89) | 0.003 |
| **Alcohol** |  |  |  |  |  |  |
| Not drink | 1.00 (reference) | - | 1.00 (reference) | - | 1.00 (reference) | - |
| Former/current drink | 1.95 (1.52-2.50) | <0.001 | 1.66 (1.27-2.16) | <0.001 | 1.60 (1.23-2.09) | 0.001 |
| **Tea drinking frequency** |  |  |  |  |  |  |
| Not drink | 1.00 (reference) | - | 1.00 (reference) | - | 1.00 (reference) | - |
| Former/current drink | 1.35 (1.11-1.65) | 0.003 | 1.30 (1.07-1.59) | 0.009 | 1.32 (1.08-1.61) | 0.007 |
| **Tea temperature ^b^** |  |  |  |  |  |  |
| Warm | 1.00 (reference) | - | 1.00 (reference) | - | 1.00 (reference) | - |
| Hot/burning hot tea | 1.32 (0.89-1.96) | 0.167 | 1.33 (0.90-1.98) | 0.151 | 1.34 (0.90-1.99) | 0.151 |
| **Source of drinking water** |  |  |  |  |  |  |
| Tap/pure water | 1.00 (reference) | - | 1.00 (reference) | - | 1.00 (reference) | - |
| Well water and surface water | 1.50 (1.24-1.81) | <0.001 | 1.63 (1.35-1.97) | <0.001 | 1.55 (1.28-1.89) | <0.001 |
| **Drink improved water** |  |  |  |  |  |  |
| No | 1.00 (reference) | - | 1.00 (reference) | - | 1.00 (reference) | - |
| Yes | 0.77 (0.51-1.11) | 0.184 | 0.74 (0.49-1.08) | 0.139 | 0.79 (0.52-1.15) | 0.251 |
| **Poultry meat** |  |  |  |  |  |  |
| No | 1.00 (reference) | - | 1.00 (reference) | - | 1.00 (reference) | - |
| Yes | 0.91 (0.74-1.12) | 0.374 | 0.74 (0.60-0.90) | 0.003 | 0.89 (0.72-1.10) | 0.290 |
| **Spring onion/ginger/garlic** |  |  |  |  |  |  |
| No | 1.00 (reference) | - | 1.00 (reference) | - | 1.00 (reference) | - |
| Yes | 0.83 (0.68-1.01) | 0.056 | 0.77 (0.64-0.94) | 0.010 | 0.85 (0.70-1.04) | 0.116 |
|  |  |  |  |  |  |  |
| The results of unconditional multivariable logistic analysis for high-grade intraepithelial neoplasia and above (continued) | | | | | | |
| **Factors** | **Age-adjusted**  **OR (95% CI)** | ***P* value** | **Gender-adjusted**  **OR (95% CI)** | ***P* value** | **Adjusted OR ^a^**  **(95% CI)** | ***P* value** |
| **Fruits** |  |  |  |  |  |  |
| No | 1.00 (reference) | - | 1.00 (reference) | - | 1.00 (reference) | - |
| Yes | 0.81 (0.67-0.99) | 0.035 | 0.74 (0.61-0.89) | 0.002 | 0.84 (0.69-1.02) | 0.085 |
| **Leftovers** |  |  |  |  |  |  |
| No | 1.00 (reference) | - | 1.00 (reference) | - | 1.00 (reference) | - |
| Yes | 1.11 (0.92-1.35) | 0.267 | 1.18 (0.98-1.42) | 0.088 | 1.16 (0.96-1.41) | 0.125 |
| **Diet taste** |  |  |  |  |  |  |
| Light diet | 1.00 (reference) | - | 1.00 (reference) | - | 1.00 (reference) | - |
| Salty diet | 1.38 (1.08-1.79) | 0.012 | 1.39 (1.09 -1.80) | 0.010 | 1.36 (1.07-1.77) | 0.016 |
| **Refrigerator** |  |  |  |  |  |  |
| No refrigerator | 1.00 (reference) | - | 1.00 (reference) | - | 1.00 (reference) | - |
| 1-10years | 0.76 (0.57-1.04) | 0.078 | 0.58 (0.43-0.79) | <0.001 | 0.76 (0.57-1.04) | 0.074 |
| 11-20years | 0.71 (0.48-1.04) | 0.076 | 0.52 (0.35-0.76) | 0.001 | 0.71 (0.49-1.05) | 0.087 |
| >20years | 0.56 (0.14-1.54) | 0.336 | 0.41 (0.10-1.13) | 0.139 | 0.55 (0.13-1.52) | 0.319 |
| **Cooking** |  |  |  |  |  |  |
| No | 1.00 (reference) | - | 1.00 (reference) | - | 1.00 (reference) | - |
| Yes | 0.79 (0.64-0.96) | 0.018 | 1.26 (0.98-1.61) | 0.072 | 1.20 (0.93-1.53) | 0.157 |
| **Housework** |  |  |  |  |  |  |
| No | 1.00 (reference) | - | 1.00 (reference) | - | 1.00 (reference) | - |
| <8h/week | 1.01 (0.74-1.39) | 0.936 | 1.10 (0.80-1.51) | 0.561 | 1.11 (0.81-1.52) | 0.531 |
| 8-14h/week | 0.82 (0.59-1.13) | 0.220 | 0.99 (0.71-1.37) | 0.932 | 0.97 (0.70-1.34) | 0.845 |
| 15-21h/week | 0.78 (0.55-1.12) | 0.179 | 1.05 (0.73-1.52) | 0.792 | 0.95 (0.66-1.38) | 0.796 |
| ≥22h/week | 0.64 (0.43-0.94) | 0.024 | 0.88 (0.59-1.32) | 0.536 | 0.79 (0.53-1.18) | 0.249 |
| **Nap** |  |  |  |  |  |  |
| No | 1.00 (reference) | - | 1.00 (reference) | - | 1.00 (reference) | - |
| Yes | 0.88 (0.72-1.08) | 0.207 | 0.80 (0.65-0.97) | 0.023 | 0.86 (0.71-1.05) | 0.140 |
| **Number of teeth lost** |  |  |  |  |  |  |
| Never | 1.00 (reference) | - | 1.00 (reference) | - | 1.00 (reference) | - |
| 1-3 | 0.84 (0.66-1.07) | 0.161 | 1.07 (0.84-1.35) | 0.599 | 0.86 (0.68-1.09) | 0.210 |
| 4-6 | 1.08 (0.81-1.44) | 0.586 | 1.69 (1.28-2.24) | <0.001 | 1.11 (0.83-1.48) | 0.484 |
| 7-11 | 0.81 (0.54-1.21) | 0.301 | 1.57 (1.05-2.35) | 0.028 | 0.83 (0.55-1.25) | 0.382 |
| 12-31 | 1.19 (0.79-1.81) | 0.409 | 2.60 (1.72-3.91) | <0.001 | 1.21 (0.79-1.84) | 0.385 |
| Complete denture | 1.16 (0.74-1.80) | 0.519 | 2.79 (1.81-4.30) | <0.001 | 1.20 (0.77-1.87) | 0.420 |
| **Loose teeth** |  |  |  |  |  |  |
| No | 1.00 (reference) | - | 1.00 (reference) | - | 1.00 (reference) | - |
| Yes | 1.53 (1.03-2.19) | 0.026 | 1.80 (1.21-2.56) | 0.002 | 1.48 (0.99-2.12) | 0.042 |
| **History of chronic hepatitis** |  |  |  |  |  |  |
| No | 1.00 (reference) | - | 1.00 (reference) | - | 1.00 (reference) | - |
| Yes | 1.94 (1.05-3.27) | 0.020 | 1.90 (1.03-3.18) | 0.024 | 1.91 (1.03-3.22) | 0.025 |
| The results of unconditional multivariable logistic analysis for high-grade intraepithelial neoplasia and above (continued) | | | | | | |
| **Factors** | **Age-adjusted**  **OR (95% CI)** | ***P* value** | **Gender-adjusted**  **OR (95% CI)** | ***P* value** | **Adjusted OR ^a^**  **(95% CI)** | ***P* value** |
| **Family history of cancer** |  |  |  |  |  |  |
| No | 1.00 (reference) | - | 1.00 (reference) | - | 1.00 (reference) | - |
| Yes | 1.59 (1.31-1.94) | <0.001 | 1.49 (1.22-1.81) | <0.001 | 1.64 (1.34-2.00) | <0.001 |
| **Take acid suppressants** |  |  |  |  |  |  |
| No | 1.00 (reference) | - | 1.00 (reference) | - | 1.00 (reference) | - |
| Yes | 0.70 (0.42-1.08) | 0.134 | 0.62 (0.37-0.97) | 0.051 | 0.68 (0.41-1.07) | 0.119 |
| **Take antibiotics** |  |  |  |  |  |  |
| Not take | 1.00 (reference) | - | 1.00 (reference) | - | 1.00 (reference) | - |
| Not every week | 0.32 (0.08-1.29) | 0.108 | 0.27 (0.07-1.09) | 0.067 | 0.34 (0.08-1.36) | 0.127 |
| Not every day | 0.79 (0.20-3.20) | 0.740 | 0.66 (0.16-0.27) | 0.555 | 0.81 (0.20-3.28) | 0.764 |
| Every day | 0.30 (0.04-2.15) | 0.232 | 0.24 (0.03-1.70) | 0.152 | 0.28 (0.04-1.99) | 0.202 |

**Legend:** ^a^ Adjusted for age, gender, education, BMI and income. ^b^ = Only part of the data with tea drinking temperature was analyzed, not all. HGIN = high-grade intraepithelial neoplasia; ESCC = esophageal squamous cell carcinoma.
